# Releasing YAP dysfunction‐caused replicative toxicity rejuvenates mesenchymal stem cells

**DOI:** 10.1111/acel.13913

**Published:** 2023-06-20

**Authors:** Fanyuan Yu, Lin Yao, Feifei Li, Chenglin Wang, Ling Ye

**Affiliations:** ^1^ State Key Laboratory of Oral Diseases & National Clinical Research Center for Oral Diseases West China Hospital of Stomatology, Sichuan University Chengdu China; ^2^ Department of Endodontics, West China Hospital of Stomatology Sichuan University Chengdu China

**Keywords:** aging, DNA damage, mesenchymal stromal cell, rejuvenation, replicative stress

## Abstract

Hippo‐independent YAP dysfunction has been demonstrated to cause chronological aging of stromal cells by impairing the integrity of nuclear envelope (NE). In parallel with this report, we uncover that YAP activity also controls another type of cellular senescence, the replicative senescence in in vitro expansion of mesenchymal stromal cells (MSCs), but this event is Hippo phosphorylation‐dependent, and there exist another NE integrity‐independent downstream mechanisms of YAP. Specifically, Hippo phosphorylation causes reduced nuclear/active YAP and then decreases the level of YAP protein in the proceeding of replicative senescence. YAP/TEAD governs RRM2 expression to release replicative toxicity (RT) via licensing G1/S transition. Besides, YAP controls the core transcriptomics of RT to delay the onset of genome instability and enhances DNA damage response/repair. Hippo‐off mutations of YAP (YAP^S127A/S381A^) satisfactorily release RT via maintaining cell cycle and reducing genome instability, finally rejuvenating MSCs and restoring their regenerative capabilities without risks of tumorigenesis.

## Graphical abstract

Fanyuan et al. revealed that YAP dysfunction occurred in the proceeding of MSCs expansion, which was the crucial driver of cellular senescence. YAP dysfunction reduced the regulatory transcriptome of cell cycle to arrest cells into G1 phase. This cycle arrest subsequently caused inevitable genome instability and impairment of DNA damage response, defined as replicative toxicity (RT). YAPS127A/S381A double‐mutation successfully prevented replicative senescence via releasing YAP dysfunction‐caused RT, and finally rejuvenated MSCs to sufficiently restore their regenerative capabilities.

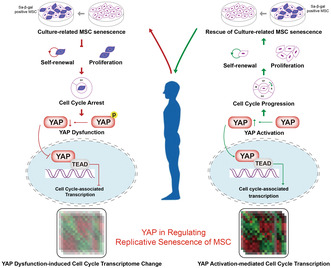

**Abbreviations**
CFM: cytoflowmetry
hDPSCs: human dental pulp‐derived stromal cells
KEGG: Kyoto Encyclopedia of Genes and Genomes
MSCs: mesenchymal stromal cells
NE: nuclear envelope
RNA‐seq: RNA‐sequencing
RT: replicative toxicity
SA‐β‐gal: senescence‐Associated β‐Galactosidase
SASP: senescence‐associated secretory phenotypes

## 1 INTRODUCTION

YAP, the core effector of hippo pathway, is recently reported to be involved in cell aging (Fu et al., 2019; Xie et al., 2013; Yu et al., 2015). As a transcription co‐activator, YAP, instead of binding DNA directly, regulates target gene expression by interaction with transcription factors such as TEADs, Smad, and Runx1/2 (Fu et al., 2019; Xie et al., 2013; Yu et al., 2015). Phosphorylated YAP is retained in cytoplasm to be utterly degraded, which leads to transcription repression of target genes (Yu et al., 2015). The relationship between YAP and cellular senescence is first reported in tumor cells (Yu et al., 2015). Senile tumor cells show less expression of YAP on both mRNA and protein levels (Yu et al., 2015). YAP dysfunction also reduces CDK6 expression in tumor cells, thereby leading to cell senescence (Xie et al., 2013). On the contrary, overexpression of active YAP is reported to alleviate chondrogenic progenitor senescence and osteoarthritis in vivo (Fu et al., 2019). YAP is furthermore revealed to be the downstream target of APC/C^Cdh1^ to control cell‐cycle progression (Kim et al., 2019). A recent study reports that YAP/TAZ activity in stromal cells prevents aging by controlling cGAS–STING (Sladitschek‐Martens et al., 2022). In parallel with this study, we also uncovered the intriguing roles of YAP in dental mesenchymal stromal cells (MSCs) senescence. In the published study by Piccolo group, authors found reduced nuclear level but unchanged phosphorylation of YAP in physiological aging of stromal cells, which was not observed in epithelial cells (Sladitschek‐Martens et al., 2022). It indicated the alteration of YAP activity differed in epithelial and mesenchymal tissues (Sladitschek‐Martens et al., 2022). Mechanistically, authors identified a canonical Hippo‐independent way via nuclear envelope (NE)‐associated cGAS–STING signaling (Sladitschek‐Martens et al., 2022). But notably, the expression of YAP^S127A^ alleviated the senescent phenotypes, indicating a controversial conclusion of Hippo phosphorylation‐linked (Sladitschek‐Martens et al., 2022). Up to date, all current reports focus on investigating the role of YAP in chronological and pathological senescence (Sladitschek‐Martens et al., 2022; Xie et al., 2013; Yu et al., 2015), but it is unknown if YAP is involved in regulating another classic type of cellular senescence, namely replicative aging in culture. In this study, we elucidated the crucial role of YAP in alleviating replication stress‐associated MSCs aging.

Nontumorous human somatic cells cannot divide indefinitely both in vivo and in vitro, which attribute to replicative senescence (Campisi, 1997; Hu et al., 2021). Replicative senescence is proposed by Hayflick et al. in 1961, defined as cells permanently out of cell cycle coupling with no responses to growth signals (Campisi, 1997; Hayflick & Moorhead, 1961; Hu et al., 2021). Recent studies furthermore indicated that chronological aging did not significantly influence the therapeutic effects of MSCs, it was replicative senescence that critically restrained their therapeutic applications (Andrzejewska et al., 2019). Although replicative senescence heavily impairs MSCs functions, their therapeutic applications always request adequate cell numbers, which requires a period of in vitro culture (Trounson & McDonald, 2015). This dilemma calls for solutions to prevent/alleviate replicative senescence of MSCs in culture and meanwhile guarantee their therapeutic efficacy and safety.

In this study, we utilized human dental pulp‐derived stromal cells (hDPSCs) as the model to investigate underlying involvement of YAP in replicative senescence. Autologous MSCs have no ethics problems and a lower risk of teratoma and immune exclusion compared to embryonic stem cells and induced pluripotent stem cells (Ben‐David & Benvenisty, 2011). MSCs can be isolated from diverse tissues, such as dental pulp, bone marrow, adipose tissue, and so on. Among heterogeneous types of MSCs, hDPSCs have various advantages such as availability (Gronthos et al., 2000), enriched stem cell numbers in the population (Alge et al., 2010), and high proliferation capabilities (Liu et al., 2015). Besides, recent comparative research has proved that hDPSCs owned substantially better regenerative and therapeutic potentials than bone marrow MSCs (Shen et al., 2019). These facts permit clinicians to have a passion for dental pulp‐derived MSCs in future regenerative medicine (Nakashima et al., 2017; Potdar & Jethmalani, 2015). Encouragingly, several pilot clinical trials demonstrate safety and efficacy of hDPSCs in regenerating dental tissues (Nakashima et al., 2017; Potdar & Jethmalani, 2015). Despite these rapid advances in MSCs‐based regenerative therapies, long‐term expansion‐related cell senescence severely retards the clinical utilization of MSCs (Alge et al., 2010; Ben‐David & Benvenisty, 2011; Gronthos et al., 2000; Liu et al., 2015; Samsonraj et al., 2017). To obtain adequate numbers of MSCs for therapy, in vitro expansion is indispensable. However, culture‐related MSCs senescence occurs along with in vitro expansion (Andrzejewska et al., 2019). This kind of cellular aging severely undermines MSCs' regenerative capabilities and limits the progression of MSCs‐based clinical utilizations (Trounson & McDonald, 2015; Truong et al., 2019; Yang et al., 2018). Dramatically decreased proliferation and impaired differentiation abilities are observed in replicatively senescent MSCs, along with apparent changes in cell morphology, metabolism, stemness, and so on (Bork et al., 2010; Truong et al., 2019; Yang et al., 2018). Especially, proliferation impairment is one of the most severe but unsolved limitations up to date. Previous reports have exhibited various characteristics of in vitro expansion‐related proliferation impairments, including cell cycle arrest, telomere change, DNA damage increment, mitochondria dysfunction, and somatic mutations (Izadpanah et al., 2008; Narisu et al., 2019; Passos et al., 2007; Yao et al., 2020). Despite these findings, molecular mechanisms preventing replicative senescence are still lacking.

In this study, we revealed that YAP dysfunction occurred in the proceeding of hDPSCs expansion, which was the crucial driver of cellular senescence. YAP dysfunction reduced the regulatory transcriptome of cell cycle to arrest cells into G1 phase. This cycle arrest subsequently caused inevitable genome instability and impairment of DNA damage response, defined as replicative toxicity (RT). YAP^S127A/S381A^ double‐mutation (YAP 2SA) successfully prevented replicative senescence via releasing YAP dysfunction‐caused RT, and finally rejuvenated hDPSCs to sufficiently restore their regenerative capabilities. These findings endow us with knowledge about the role of YAP in replicative senescence and methods to solve current limitations of MSCs‐based regenerative applications.

## 2 RESULTS

### 2.1 RT emerges upon in vitro expansion of dental MSCs

hBMSCs and hDPSCs are often compared as they share similarities and meanwhile own disparities (Shen et al., 2019). Our data showed that upon in vitro expansion, the proliferation inhibition occurred much earlier in hBMSCs than hDPSCs (Figure S1A). Accordingly, such early appeared replication stress was companioned with cellular senescence in synchrony in hBMSCs but not in hDPSCs (Figure S1B). These data showed that hDPSCs owned a stronger capability to resist replicative senescence than hBMSCs. Sequential data of in vitro expansion yet demonstrated that even for hDPSCs, replicative senescence and severely impaired stemness were inevitable (Figure S1C). Specifically, cytoflowmetry (CFM) furthermore revealed that replicatively senescent hDPSCs were arrested at G1 phase but not G2 (Figure S1D). G1‐arrested hDPSCs demonstrated increased genome instability, reduced stemness markers, obvious senescence‐associated secretory phenotypes (SASP), and increased cell size (Figure S1E–H). These data revealed that even hDPSCs showed an advantage of delayed happening of replicative senescence upon in vitro expansion, but RT phenotypes were still inevitable.

### 2.2 RT owns core transcriptomic signature of 108 down‐regulated genes

Characteristics of RT include transcriptomic alterations (Shen et al., 2019). RNA‐sequencing (RNA‐seq) data showed that in hBMSCs and hDPSCs, significantly altered genes happened in replicatively senescent cells, which was consistent in adolescent and adult stages (Figure S2A). The young and senile cells in this study specifically indicated the early passage and replicatively senile cells, otherwise specifically indicated. UMAP showed that gene expression profiles substantially changed in replicatively senescent MSCs without effect by biological age (Figure S2B). In the adolescent group, RT‐associated transcriptomic changes were mainly downregulated, both in hBMSCs and hDPSCs, with 185 mutually decreased genes (Figure S2C). To detect the potential influence of biological age on RT‐associated transcriptomic changes, we analyzed the RNA‐seq data of hBMSCs at different ages (Figure S2D). Results showed that in adolescent, adult, and aged hBMSCs, the top common RT‐associated transcriptomic alteration was down regulation, including 110 genes (Figure S2D). Furthermore, integrated RNA‐seq analysis of different cell types and biological ages demonstrated the core transcriptomic signature of RT in MSCs was 108 down‐regulated genes (Figure S2E). In‐depth bioinformatic analysis revealed the functions of these shared 108 down‐regulated genes were about cell cycle regulation, especially G1/S license, and DNA damage response/repair (Figure S3).

### 2.3 YAP dysfunction in protein activity positively links with the proceeding of RT

**FIGURE 1:**
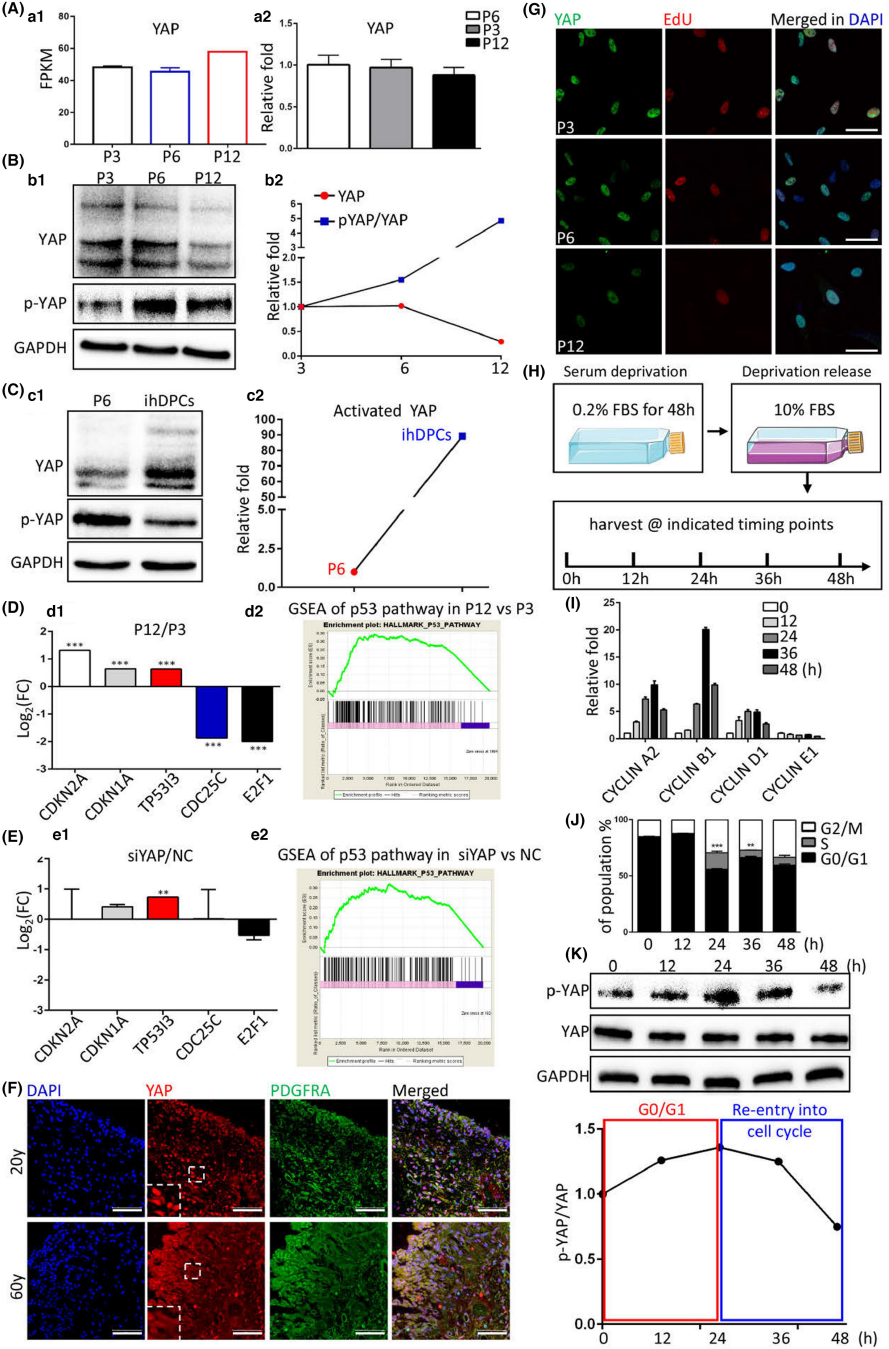
YAP dysfunction in protein activity positively links with the proceeding of RT. (A) RNA‐seq data showing the Fragments Per Kilobase of exon model per Million mapped fragments (FPKM) of YAP (a1), and confirmative RT‐qPCR data of YAP (a2). (B, C) Representative western blotting images (b1 & c1) and their statistical data (b2 & c2). p‐YAP indicated S127‐phosphorylated YAP. (D, E) Statistical data of RNA‐seq (d1 & e1), and according Gene Set Enrichment Analysis (GSEA) analysis of P53 pathway (d2 & e2). (F) Immunofluorescence (IF) images of human molar pulp. Scale bars, 50 μm. (G) Combined IF of YAP and EdU incorporation data. Scale bars, 10 μm. (H) Schematic illustration of serum deprivation synchronization model. (I) RT‐qPCR data showing the changes of genes at the harvest timing points of (H). (J) The statistical data of FCT of cell cycle. (K) Representative western blotting images and their statistical data. p‐YAP indicated S127‐phosphorylated YAP. **p < 0.01; ***p < 0.001. All experiments were technically replicated for triple times.

YAP has already been reported to regulate cellular senescence via controlling gene expressions (Fu et al., 2019; Kim et al., 2019; Sladitschek‐Martens et al., 2022; Yu et al., 2015), which contain the main cell cycle and DNA damage responses/repair genes identified by us (Figure S2). Therefore, we firstly ascertained whether YAP mediated the core transcriptomic signature of RT. RNA‐seq and RT‐qPCR data of hDPSCs in the proceeding of in vitro expansion showed no obvious change in YAP mRNA expressions (Figure 1A). But the pan‐YAP protein was substantially reduced at P12, along with the activity of YAP continuously weakened from P6 (Figure 1B). In our previously reported hDPSCs cell line (ihDPSCs) (Li et al., 2020), which demonstrated actively cycling characteristic, both the total YAP protein level and YAP activity were notably higher than primary cultured hDPSCs (Figure 1C). Furthermore, RNA‐seq data showed that the reduced transcriptomic hallmarks of RT (Figure 1D) were partially reappeared after YAP knockdown (Figure 1E), showing clues that YAP activity in hDPSCs probably in charge of RT‐associated core transcriptome. In addition to replicative senescence, our data showed that in chronological ageing, nuclear localization of YAP was also decreased in aged hDPSCs (Figure S4A,B and Figure 1F). It indicated that YAP dysfunction was a mutual mark in replicative and biological senescence of hDPSCs. Furthermore, EdU incorporation experiment revealed that along with in vitro expansion, reduced nuclear YAP positively linked with decreased mitosis (Figure 1G). Taken the transcriptomic effect of YAP on regulating RT‐associated cell cycle genes (Figure 1D,E) together, we sought to detect if YAP activity influence RT‐associated G1 arrest. We established a serum deprivation method to synchronize hDPSCs out of cell cycle (Figure 1H), and then release deprivation to make cells reenter into cell cycle (Figure 1H–J). Data revealed that after reentry into cell cycle YAP activity continuously rose, which began at G1/S transition (Figure 1K). EdU incorporation furthermore proved that losing YAP activity in hDPSCs made cells unable to enter into cell cycle (Figure S4C).

### 2.4 YAP dysfunction reappears RT‐associated transcriptomic alterations in cell cycle and DNA damage response

**FIGURE 2:**
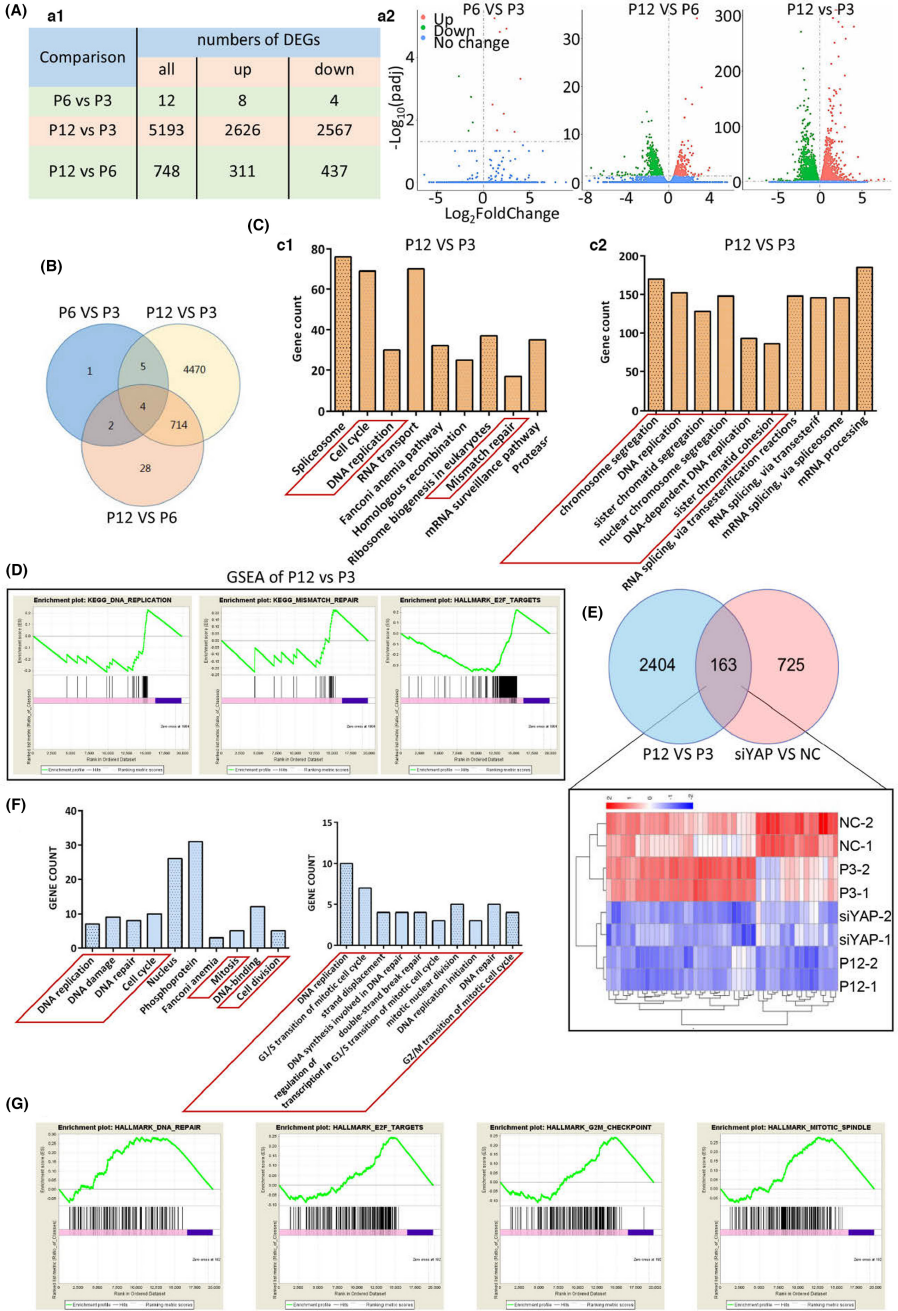
YAP dysfunction reappears RT‐associated transcriptomic alterations in cell cycle and DNA damage response. (A) The summary of the DEG numbers (a1) and the volcano plot (a2) of RNA‐seq. (B) Venn diagram of DEGs in RNA‐seq datasets. (C) KEGG (c1) and geneontology (GO‐term) analysis of down‐regulated DEGs in comparison between P12 and P3. (D) GSEA analysis of down‐regulated DEGs in comparison between P12 and P3, respectively showing DNA replication, mismatch repair, and E2F hallmarks. (E) Venn diagram (up) and heatmap (bottom) showing the down‐regulated DEGs in two pairs of comparison: P12 vs. P3 and siYAP versus negative control (NC). (F) KEGG (left) and GO‐term (right) analysis of the 163 shared down‐regulated DEGs in (E). (G) GSEA analysis of 163 shared down‐regulated DEGs in (E), respectively showing hallmarks of DNA repair, E2F hallmarks, G2/M checkpoint, and hallmarks of mitotic spindle. *p < 0.05; **p < 0.01; ***p < 0.001. All experiments were technically replicated for triple times.

Following the clues that YAP activity in hDPSCs was probably in charge of RT‐associated core transcriptome (Figure 1D,E), we investigated this hypothesis in details. Transcriptomic alterations in the proceeding of in vitro expansion of hDPSCs did not occur gradually (Figure 2A). From P3 to P6, minimal genes were differentially expressed, but at P12, the transcriptomic alterations boomed (Figure 2A,B and Figure S5B). This finding was consistent with our finding that at P6 no obvious cellular senescence was observed (Figure S1C c1 & 5). Functional enrichment and GSEA analysis of P12 vs. P3 showed that the down‐regulated genes in RT were concentrated within cell cycle regulation and DNA damage response/repair (Figure 2C,D). Loss of YAP in young hDPSCs caused significantly differential gene expressions (Figure 2E), and the majority was down‐regulated (Figure S5C). Venn diagram and Heatmap analysis showed that knockdown of YAP and RT owned 163 mutually reduced genes (Figure 2E). Functional enrichment and GSEA analysis of these 163 genes demonstrated the YAP dysfunction reappeared the RT‐associated transcriptomic alterations in inhibiting cell cycle and DNA damage response/repair (Figure 2F,G).

### 2.5 YAP activity guarantees G1/S transition to prevent RT

**FIGURE 3:**
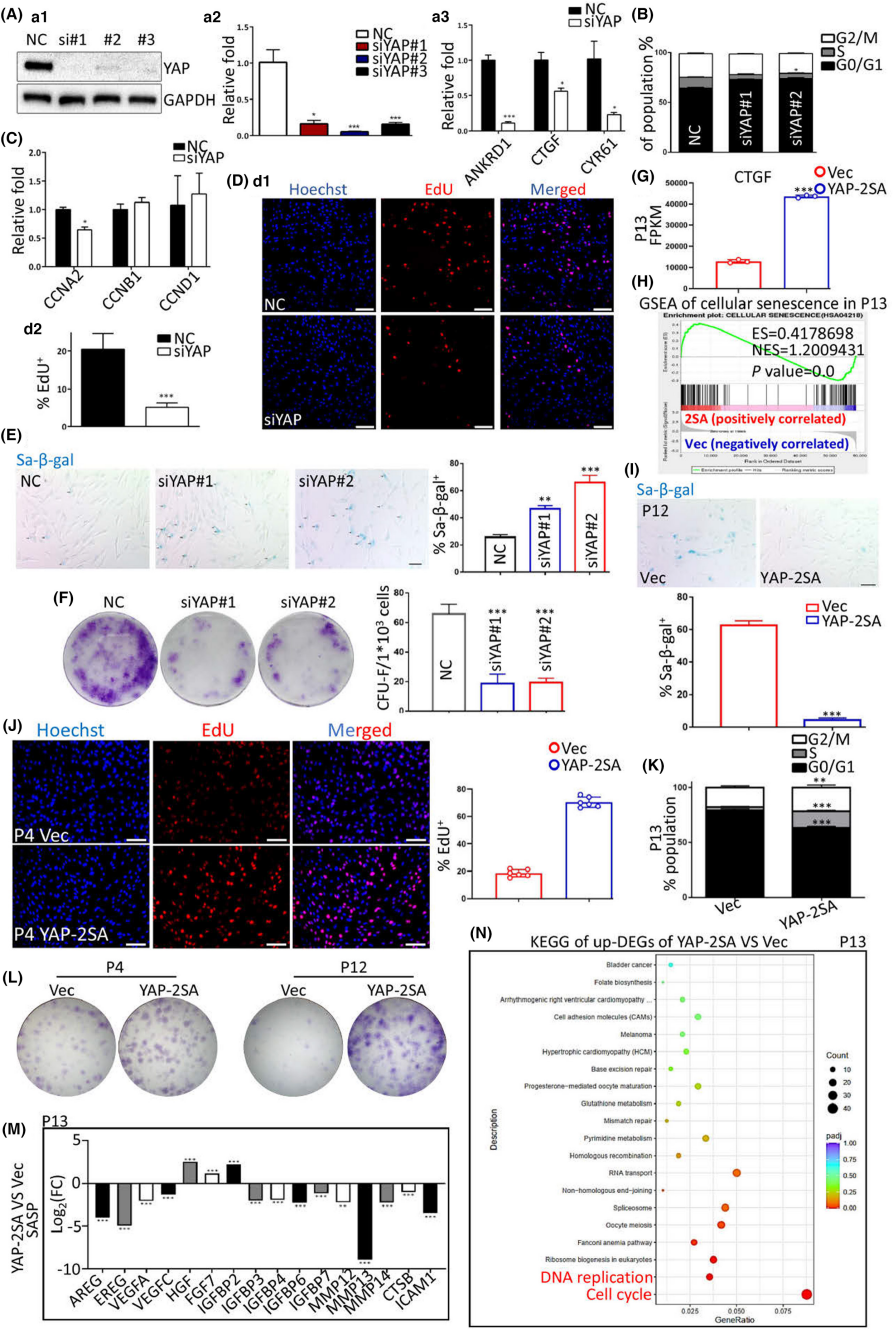
YAP activity guarantees G1/S transition to prevent RT. (A) Representative western blotting image (a1) and its statistical data (a2), and the RT‐qPCR data of YAP‐downstream genes (a3). *n* = 3 per group. (B) The statistical data of FCT of cell cycle. *n* = 3 per group. (C) RT‐qPCR data. (D) Representative image of EdU incorporation (d1) and its statistical data (d2). *n* = 3 per group. (E) Representative SA‐β‐gal staining and its statistical data. (F) Representative CFU data and its statistical results. (G) RNA‐seq data showing the FPKM values of YAP‐downstream gene CTGF. *n* = 3 per group. (H) GSEA analysis of cellular senescence pathway according to RNA‐seq data of YAP‐2SA expressed hDPSCs. (I) Representative SA‐β‐gal staining and its statistical data. (J) Representative image of EdU incorporation and its statistical data. *n* = 6 per group. (K) The statistical data of FCT of cell cycle. *n* = 3 per group. (L) Representative CFU images. (M) The gene set of SASP according to the statistical data of RNA‐seq. FC, fold change. (N) KEGG analysis of up‐regulated DEGs in P13 according to RNA‐seq. *p < 0.05; **p < 0.01; ***p < 0.001. Scale bars, 15 μm. All experiments were technically replicated for triple times.

To ascertain the exact role of YAP in RT‐associated G1 arrest, we investigated this aspect as follows. After confirming the knockdown of YAP and the loss of YAP activity (Figure 3A), we observed that YAP dysfunction phenocopied the G1 arrest (Figure 3B,C), which caused inevitable proliferation inhibition (Figure 3D and Figure S4C) and finally led to cellular senescence and stemness loss (Figure 3E,F). When we put YAP^S127/381A^ double‐mutation (YAP 2SA) into hDPSCs, (the Hippo phosphorylation‐deficient continuous activation form of YAP) (Figure 3G), we observed significantly inhibited cellular senescence in transcriptomics (Figure 3H) and YAP 2SA satisfactorily rejuvenated senescent hDPSCs (Figure 3I). YAP 2SA at P4 significantly enhanced the mitosis of hDPSCs (Figure 3J), which caused concerns about tumorigenesis risks. RNA‐seq data and in vivo transplantation results showed that YAP 2SA in hDPSCs did not induce tumors as most crucial oncogenes were downregulated (Figure S6). After confirming the safety of YAP 2SA in hDPSCs, we uncovered that YAP 2SA significantly reduced G1‐arrested cells at P13 (Figure 3K). Increased G1/S transition in the YAP 2SA group helped hDPSCs reenter mitosis (Figure 3K). Such relieved proliferation inhibition restored the self‐renewal capability of hDPSCs (Figure 3L), and meanwhile alleviated SASP (Figure 3M). Kyoto Encyclopedia of Genes and Genomes (KEGG) analysis of P13 hDPSCs showed that YAP 2SA substantially up‐regulated cell cycle maintenance and DNA damage response/repair genes (Figure 3N). The top transcriptomic functions regained by YAP 2SA were for guaranteeing mitosis and genome stability (Figure 3N and Figure S7), and meanwhile, cellular senescence was alleviated (Figure S7), showing that YAP 2SA prevented RT via guaranteeing G1/S transition.

### 2.6 The regulatory effects of YAP in RT depend on RRM2 transcription

**FIGURE 4:**
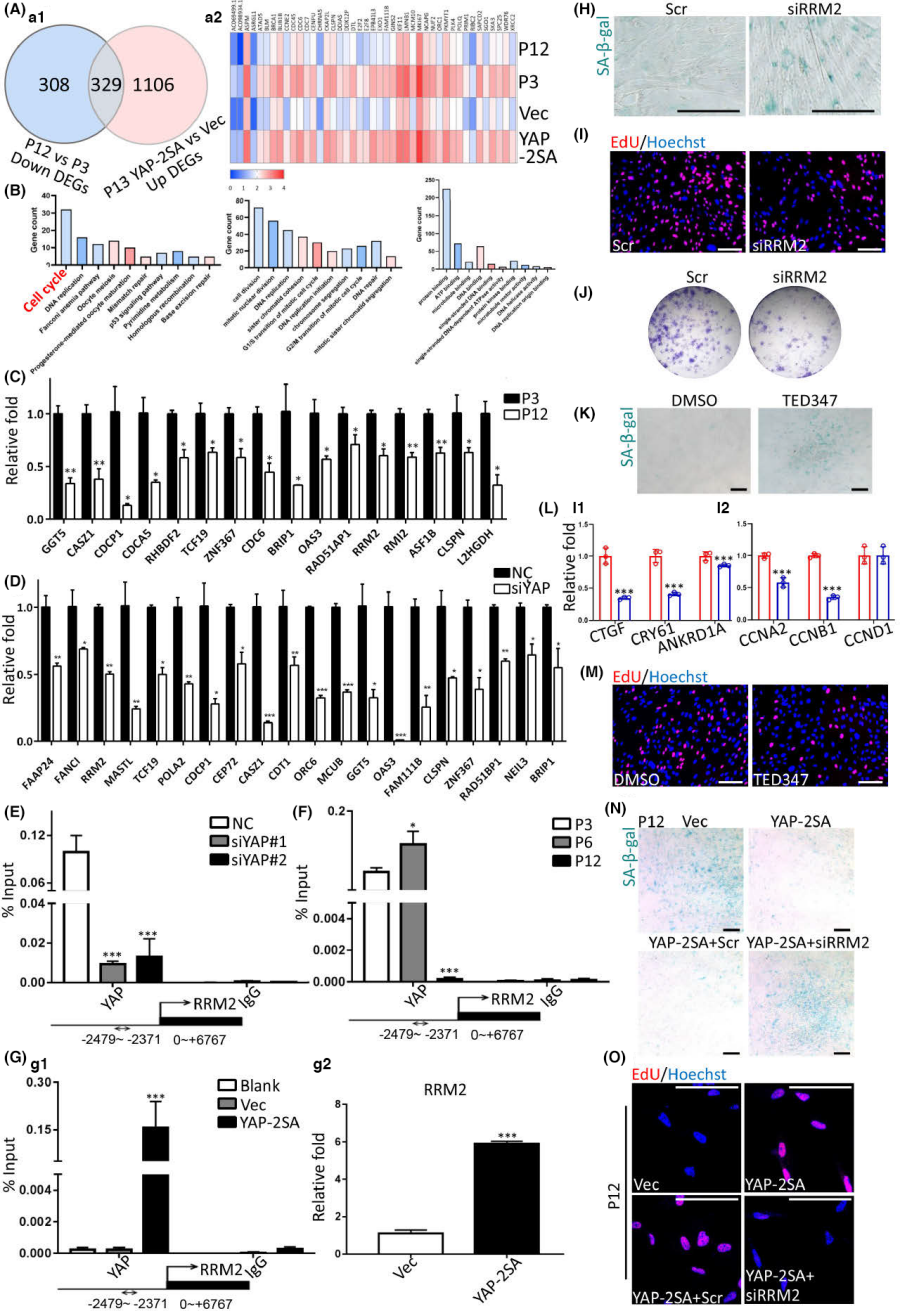
The regulatory effects of YAP in RT depend on RRM2 transcription. (A) The Venn diagram (a1) and heatmap (a2) showing the shared DEGs in following comparison pairs, namely the down‐regulated DEGs between P12 vs. P3 and the up‐regulated DEGs between YAP‐2SA versus control (Vec) in P13. (B) The KEGG (left), GO‐term of biological process (middle), and GO‐term of molecular function (right) analysis of the 329 common DEGs in (A). (C, D) The confirmative RT‐qPCR data of the down‐regulated genes identified in RNA‐seq. *n* = 3 per group. (E, F) ChIP‐qPCR data showing the enrichment of YAP protein within the promoter regions of RRM2 gene. *n* = 3 per group. (G) ChIP‐qPCR data showing the enrichment of YAP protein within the promoter regions of RRM2 gene (g1) and the RT‐qPCR data of RRM2 (g2). *n* = 3 per group. (H–J) Representative SA‐β‐gal staining (H), EdU incorporation (I), and CFU data (J) after siRRM2 at P4. (K) Representative SA‐β‐gal staining after using TED347 at P4. (L) RT‐qPCR data of YAP downstream genes (l1) and cycle regulators (l2). *n* = 3 per group. (M) Representative EdU incorporation data after using TED347 at P4. (N, O) Representative SA‐β‐gal staining (N) and EdU incorporation (O). *p < 0.05; **p < 0.01; ***p < 0.001. Scale bars, 15 μm. All experiments were technically replicated for triple times.

Integrated RNA‐seq analysis showed that RT‐associated down‐regulated transcriptomics shared 329 genes up‐regulated by YAP 2SA (Figure 4A). Functional enrichment of these 329 genes demonstrated that YAP activity mainly restored the impaired functions of cell cycle maintenance and DNA damage response/repair of RT (Figure 4B). Next, we utilized RT‐qPCR and confirmed the top reduced cell cycle maintenance and DNA damage response/repair genes, which were respectively identified by RNA‐seq of RT and YAP knockdown (Figure 4C,D). RT‐qPCR data identified the YAP‐dependent genes which probably prevented RT (Figure 4C,D and Figures S8 and S9). As data of YAP 2SA confirmed that YAP function in regulating hDPSCs' RT was Hippo phosphorylation‐dependent (Figure 3), we hypothesized whether the downstream effect of YAP also followed the canonical Hippo transduction. To investigate this, we first screened the putative downstream genes of TEAD4 in RT of hDPSCs, to ascertain the possibility that they were YAP/TEAD‐mediated transcriptions (Figures S8 and S9). ChIP‐seq data showed that among these genes, RRM2 was the promising candidate for YAP/TEAD complex (Figure S8). In details, the promoter region of RRM2 was highly occupied with TEAD4, and accordingly, the active epigenetic modifications of H3K4me3 and H3K27Ac were highly enriched within the same regions (Figure S8). Next, we used ChIP‐qPCR to furthermore analyze the TEAD4 binding region of RRM2 identified by ChIP‐seq. Results showed that knockdown of YAP significantly reduced the TEAD4 enrichment (Figure 4E), combined with RNA‐seq and RT‐qPCR data of RRM2 expression after silencing YAP (Figure 4D), which identified that RRM2 was the putative downstream of YAP/TEAD4 complex. Furthermore, in the proceeding of RT, such complex enrichment within RRM2's promoter also descended (Figure 4F), along with the decreased transcription of RRM2 (Figure 4C). On the contrary, putting YAP 2SA into senescent hDPSCs restored the enrichment of YAP/TEAD4 complex (Figure 4G g1), companioned with up‐regulated RRM2 expression (Figure 4G g2 and Figure S10C). Loss of RRM2 phenocopied cellular senescence, proliferation inhibition, and stemness impairment (Figure 4H–J and Figure S10A,B) as RT and YAP dysfunction in hDPSCs. The utilization of an irreversible, covalent, and allosteric inhibitor at YAP‐TEAD protein–protein interaction, namely TED 347, also caused cellular senescence in hDPSCs (Figure 4K). After confirming the inhibitory function of TED 347 (Figure 4L l1), we observed the reduced RRM2 expression (Figure S10D) and proliferation inhibition function of TED 347 (Figure 4L l1,M and Figure S10E,F). Together, current data indicated that the regulatory effects of YAP in RT might depend on RRM2 transcription. Data proved that loss of RRM2 extinguished the RT prevention and rejuvenation functions of YAP 2SA (Figure 4N,O).

### 2.7 Regaining YAP activity reduces genome instability in RT

**FIGURE 5:**
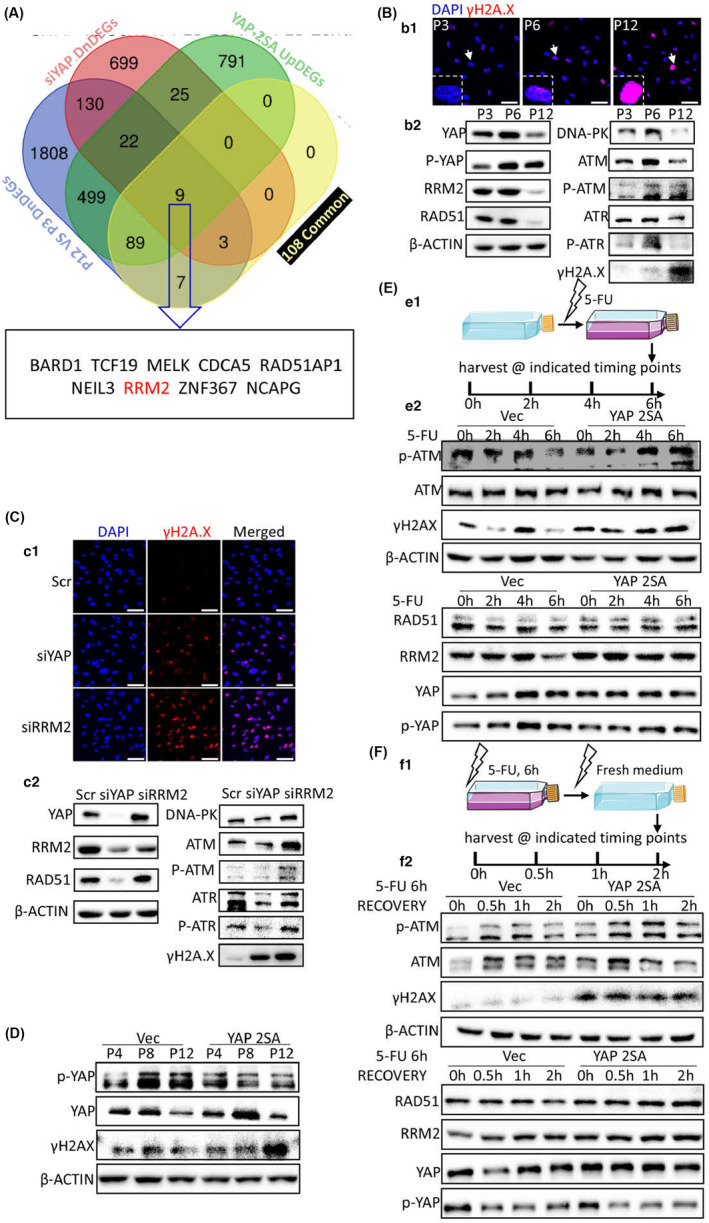
Regaining of YAP activity reduces genome instability in RT. (A) Venn diagram showing the 9 common DEGs in versatile comparison pairs according to RNA‐seq data. (B) Representative images of EdU incorporation (b1) and western blotting (b2). The white arrows in (b1) indicated the zoomed regions showed within dotted boxes. (C) Representative images of EdU incorporation (c1) and western blotting (c2). (D) Representative images of western blotting. (E) Schematic illustration of 5‐FU stress experiments (e1) and the representative images of western blotting (e2). (F) Schematic illustration of 5‐FU recovery experiments (f1) and the representative images of western blotting (f2). Scale bars, 10 μm. All experiments were technically replicated for triple times.

Integrated analysis of RNA‐seq revealed that YAP controlled nine genes of RT‐associated core transcriptomic signature from 108 down‐regulated genes for cell cycle and DNA damage regulation (Figure 5A). Together with afore discovered data, we sought to furthermore understand the mechanism by which YAP alleviated the genome instability in RT. In the proceeding of RT, the genome instability hallmark γ‐H2A.X foci continuously increased (Figure 5B b1), but the protein level of RRM2, YAP, YAP activity, and the crucial DNA damage response/repair signals, including RAD51, DNA‐PK, and ATM/ATR activity, were significantly decreased in replicative senescent hDPSCs (Figure 5B b2). It showed that replicatively senescent hDPSCs lost the capability to maintain genome stability. Data of γ‐H2A.X showed that loss of YAP or RRM2 both caused severe DNA damage (Figure 5C). YAP knockdown impaired the essential DNA damage response/repair machinery, including RAD51, ATM activation, and ATR activation (Figure 5C). But RRM2 knockdown did not show the same effect of siYAP of RAD51, ATM, and ATR (Figure 5C), showing that the cell cycle regulation of YAP was RRM2 dependent, but the DNA damage response/repair was RRM2 independent. YAP 2SA both restored YAP protein level and YAP activity, which delayed the onset of genome instability in the proceeding of RT (Figure 5D). Next, we utilized Fluorouracil (5‐FU) to analyze the underlying mechanisms. Results showed that upon 5‐FU stimulation wildtype hDPSCs demonstrated rapid DNA damage and increased genome instability within 2 h (Figure 5E). But in YAP 2SA group, the onset of DNA damage was obviously delayed at 4 h‐post 5‐FU treatment (Figure 5E). It was assumed that enhanced G1/S transition and mitotic proceeding in YAP 2SA group helped cells to dilute the toxicity of 5‐FU, but this point needs to be furthermore analyzed. For detecting DNA damage response/repair capability, we next carried out 5‐FU recovery experiment (Figure 5F f1). Results showed that in the recovery stage YAP 2SA group had stronger and faster responses to DNA damage to initiate repair (Figure 5F f2). Taken together, regaining YAP activity reduces genome instability in RT via delaying the onset of genome instability and meanwhile triggering faster and stronger capability of DNA damage response/repair.

### 2.8 Releasing YAP dysfunction‐caused RT restores regeneration

**FIGURE 6:**
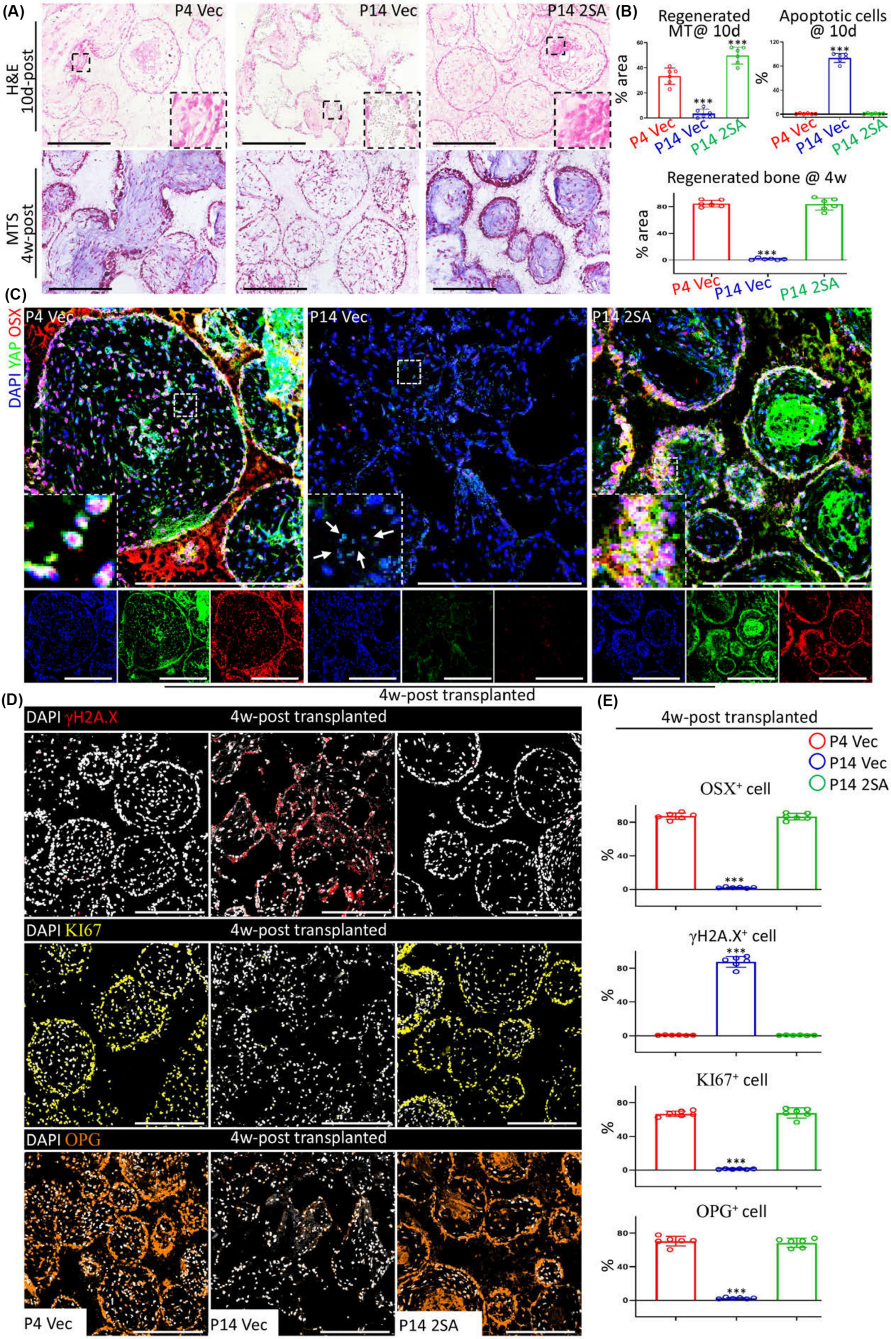
Releasing YAP dysfunction‐caused RT restores regeneration. (A) Representative images of H & E staining (up) and Masson's trichrome staining (bottom) after tissue engineering surgeries. Scale bars, 100 μm. (B) Statistical data of histological staining after tissue engineering surgeries. *n* = 6 per group. (C) Representative IF images at 4w after transplantation surgeries. Scale bars, 50 μm. (D, E) Representative IF images at 4w after transplantation surgeries (D) and its statistical data (E). Scale bars, 100 μm. *n* = 6 per group. ***p < 0.001. All experiments were technically replicated for triple times.

Tissue engineering‐based hDPSCs transplantation was carried out following our previous study (Yu et al., 2020). In vivo transplantation data showed that at 10d after transplantation, very limited cells survived within scaffolds in replicatively senescent group in comparison to young cell group (Figure 6A,B). In P14 Vec group many apoptotic cells were observed, but P4 Vec did not show such a phenotype (Figure 6A,B). Besides, prolonged follow‐up demonstrated that young hDPSCs regenerated new bone within the porous cavities of hydroxyapatite (HA) scaffolds (Figure 6A,B), but replicative senescent cells barely showed regenerated hard tissues (Figure 6A,B). However, YAP 2SA rejuvenated replicative senile hDPSCs, storing their regenerative capabilities in vivo to increase cell survival, self‐renewal, and new bone formation as well as to reduce senescence‐associated apoptosis (Figure 6A,B). Furthermore, serial assessments demonstrated that YAP 2SA successfully released YAP dysfunction‐associated RT in vivo (Figure 6C–E), which enhanced osteogenic lineage commitment and self‐renewal of hDPSCs at the late passage and reduced the genome instability (Figure 6C–E). Together with the satisfactory safety assessment of YAP 2SA in tissue engineering (Figure S6), these data proved that regaining YAP activity was the efficient and safe solution to solve the problem of expansion‐associated cellular senescence when applying hDPSCs in tissue engineering and regenerative therapies.

## 3 DISCUSSION

In parallel with the very recent publication, which revealed the inhibitory roles of YAP activity in biological aging of MSCs (Sladitschek‐Martens et al., 2022), we identified that YAP activity was crucial in preventing in vitro replicative senescence of hDPSCs too. Although for biological aging of MSCs, authors concluded that the reduced nuclear localization of YAP was canonical Hippo‐independent, their data of YAP^S127A^ mutation contradicted this conclusion (Sladitschek‐Martens et al., 2022). In this study, we found that for replicative senescence of MSCs, however, the dysfunction of YAP was upon canonical Hippo phosphorylation. First, we observed that in replicative senescence, the level of phosphorylated‐S127 YAP was increased along with reduced pan YAP protein. Secondly, YAP 2SA double‐mutation satisfactorily prevented replicative senescence and restored the regenerative capabilities of senile cells.

In this study, we revealed a different mechanistic basis of YAP‐dysfunction caused MSCs aging. For biological aging, researchers reported that reduced nuclear YAP caused the loss of NE integrity, further triggering cGAS–STING‐associated cellular senescence (Sladitschek‐Martens et al., 2022). According to our data, we cannot exclude the possible participation of this regulatory mechanism in replicative senescence. Our results demonstrated that YAP activity controlled the RT‐associated core transcriptomic, including RRM2, to maintain mitosis and reduce genome instability, specifically via guaranteeing G1/S transition. Loss of RRM2 abolished the rejuvenation effect of YAP 2SA. Besides, YAP activity delayed the onset of genome instability and meanwhile triggered faster and stronger capability of DNA damage response/repair. The YAP activity in replicative senescence of MSCs was upon the transcriptional regulation of YAP/TEAD complex. Generally, replicatively senescent cells are known to be arrested in G1 or G0 stage of the cell cycle. While a previous study reported in specific cell types shows a large fraction of replicative senescent cell population was also arrested in G2 phase (Mao et al., 2012). It is unknown before which phase is arrested in senescent MSCs, nor do we know the underlying mechanisms causing this cycle arrest in MSCs. In this study, we uncovered that replicative senescent MSCs were G1‐arrested and YAP/TEAD‐associated transcriptional regulation controlled this event.

Despite plenty of advances in MSCs‐based regenerative medicine, long‐term in vitro expansion‐induced cell senescence still significantly impedes clinical utilization of MSCs (Samsonraj et al., 2017; Trounson & McDonald, 2015). Therefore, “anti‐*in vitro* expansion‐related aging” strategies have been pursued for decades (Bork et al., 2010; Passos et al., 2007; Trounson & McDonald, 2015; Truong et al., 2019; Yang et al., 2018). Here, we discovered that regain of YAP activity during in vitro expansion was a promising way to avoid replicative senescence in hDPSCs. In this study, we reported that cell‐cycle progression‐related transcriptome was significantly decreased in replicative senescent MSCs, which continuously deteriorated during in vitro expansion. We furthermore revealed that loss of YAP function caused this kind of gene expression inhibition, next restricting G1/S transition, consequently catalyzing proliferation impairment and cellular senescence. Restoration of YAP activity alleviated replicative senescence of MSCs via delaying RT‐associated G1 arrest and genome instability. Mechanistically, we identified RRM2, a crucial G1/S licensor (Chen et al., 2014), as the direct downstream of YAP in regulating RT‐associated cell cycle arrest. Besides, Hippo‐off YAP 2SA delayed the onset of genome instability and meanwhile enhanced DNA damage response/repair, which alleviated genome instability in the proceeding of RT development. Finally, in vivo transplantation results demonstrated the satisfactory efficacy of improving YAP activity on anti‐in vitro expansion‐related aging and guaranteeing the regenerative capabilities of hDPSCs. And notably, in vitro and in vivo data evidenced the sufficient safety of rejuvenating hDPSCs via enhancing YAP activity.

In summary, our data provide novel insights into replicative senescence of MSCs. These findings endow us with a promising way to resolve the long‐standing challenge in MSCs‐based medicine and may significantly faster the application of MSCs in regenerative therapies.

## 4 MATERIALS AVAILABILITY

All unique/stable reagents generated in this study are available from Dr. Fanyuan Yu (fanyuan_yu@outlook.com) with a completed Materials Transfer Agreement.

## 5 MATERIALS AND METHODS

### 5.1 Ethics statement

All animal procedures and human dental pulp sample procedures were reviewed and approved by Ethical Committees of West China School of Stomatology, Sichuan University (WCHSIRB‐D‐2020‐393). Relative surgical models were conducted according to approved guidelines set by State Key Laboratory of Oral Diseases, West China Hospital of Stomatology.

### 5.2 Experimental procedures

#### 5.2.1 Animals

Eight weeks old Balb/c immune‐deficient nude mice used as recipients of hDPSCs subcutaneous transplantations were purchased from Dossy (Chengdu Dossy Experimental Animals Co). All animal studies conformed to ARRIVE (Animal Research: Reporting of In Vivo Experiments) guidelines. In brief, all mice were housed in SPF Experimental Animal Core of West China Hospital, Sichuan University (China), which was a temperature controlled (25°C) environment under a 12‐hour light/12‐hour dark cycle with cotton batting. The hydroxyapatite scaffold‐loaded hDPSCs transplantation strictly followed our previous published protocols (Yu et al., 2020).

#### 5.2.2 Cells

Human dental pulp‐derived MSCs were harvested in third molars from healthy donors (all cell experiments were from donors of 18–22 years old) in West China Hospital of Stomatology. All human dental pulp tissues were collected according to the informed protocol approved by the Committee on Human Research of the West China Hospital of Stomatology of Sichuan University (approval# WCHSIRB‐D‐2012‐0015). We followed the approved guidelines and obtained informed consent from donors. Primary dental pulp‐derived MSCs were cultured in α‐MEM (GIBCO) supplemented with 10% FBS (GIBCO) and 1% Penicillin–Streptomycin (GIBCO). We used P6 dental pulp‐derived MSCs for experiments which were not specifically indicated with passage in this study. The highly‐proliferative immortalized human dental pulp‐derived MSCs line (ihDPCs) was established by our collaboration lab and specifically described in previous study (Li et al., 2020). Culture of ihDPCs followed the same protocol of primary dental pulp‐derived MSCs.

#### 5.2.3 Small interfering RNA (siRNA) transfection and viral infection

Cells were transfected with 30 nmoL/L siRNAs duplex for 8 hours using Lipofectamine™ 3000 reagent (Invitrogen) following the manufacturer's protocol. The negative control RNA (NC) and YAP‐specific siRNAs were purchased from Thermo fisher scientific (YAP Stealth siRNAs ID: HSS115942, HSS115944, HSS173621; Stealth RNAi™ siRNA Negative Control Low GC Duplex #2). The detailed information of siRNAs for RRM2 was listed in Table S2. YAP^S127A/S381A^ double‐mutation (YAP‐2SA) lentiviral plasmid was constructed by site‐directed mutagenesis in accordance with previous study (Miyamura et al., 2017). YAP 2SA and vector plasmids were packaged and purified in accordance with a previously reported protocol (Jiang et al., 2015). For viral infections, before switched to growth medium cells were incubated with the viruses‐contained medium for 12 h. Then medium was changed to complete medium. Each siRNA transfection and viral infection was verified by RT‐qPCR. Upon verification of efficient infection, the cells were used for further experiments.

#### 5.2.4 Serum deprivation synchronization model

For G0/G1 synchronization, briefly, cells were cultured in 0.2% FBS medium for 48 h. And then cells were released using fresh complete expansion medium containing 10% FBS. After being released, cells were harvest at indicated times. The schematic illustration of this model was provided in Figure 5A.

#### 5.2.5 RT‐qPCR

Total RNA was extracted using TRIzol™ (Invitrogen) according to the manufacturer's protocol. Reverse transcription was performed with a PrimeScript®RT reagent kit with gDNA Eraser (TaKaRa). Quantitative real‐time PCR was carried out using a standard SYBR Green PCR Kit (TaKaRa) on a CFX96 detector (Bio‐Rad). Glyceraldehyde‐3‐phosphate dehydrogenase (GAPDH) was used to normalize the expression level of each gene. RT‐qPCR primers used in this study were provided in Table S2.

#### 5.2.6 Western blot

For western blot, cells were prepared using a protein lysis solution (Pierce Biotechnology) containing protease and phosphatase inhibitor cocktail (Millipore). Protein extraction was performed according to the manufacturer's instructions. Total cell lysates were analyzed by western blotting according to standard procedures. Proteins were visualized using ImageLab (Bio‐Rad) according to the manufacturer's instructions. All antibodies used in this study were listed in Table S1.

#### 5.2.7 Colony formation assay

Cells were seeded in a six‐well plate at a density of 1000 cells/well and cultured in growth medium for 10 days. Cells were washed with PBS, fixed in a 4% paraformaldehyde for 5 min, and then stained with crystal violet (Beyotime) for 5 min.

#### 5.2.8 Flow cytometry analysis

To analyze the cell cycle distribution, trypsinized cells were washed with PBS and fixed in 70% ethanol at 4°C overnight. Cells were then washed twice with PBS and incubated with RNase for 30 min at 37°C, followed by incubation with propidium iodide (KeyGEN Biotech) for 30 min at 4°C. The propidium iodide‐stained cells were examined on a Guava EasyCyte HT flow cytometer (Millipore) and analyzed with InCyte2.7 software (Millipore).

#### 5.2.9 Senescence‐Associated β‐Galactosidase staining (SA‐β‐gal)

Cells were seeded in a 96‐well plate at a density of 6000 cells/well and cultured in growth medium. Cells were washed in PBS, fixed in SA‐β‐gal staining‐fixture (Beyotime) for 15 min at room temperature, then washed for five times with PBS. And finally, cells were incubated with freshly prepared staining solution (Beyotime) overnight at 37°C.

#### 5.2.10 Whole‐genome RNA‐seq and bioinformatic analysis

Total mRNA of human dental pulp‐derived MSCs were collected using TRIzol™ (Invitrogen) and extraction procedures were conducted according to manufacturer's instructions for adherent cells RNA extraction. After quality checks the RNA samples were subjected to whole‐genome RNA‐seq (Novagene). Array and data were performed and analyzed by Novogene (China). Genes with an expression fold change≥2 and *p* values <0.05 were considered as significant in this study. The accession numbers of RNA‐seq in this study are GSE148287 and GSE213339. With respect to the RNA‐seq data of in vitro replicative senescence of adolescent hBMSCs were also deposited to gene expression omnibus (GEO) with accession number of GSE160273.

We downloaded the GSE35957 microarray dataset from GEO database (Benisch et al., 2012), which comprises of 10 transcriptome of bone marrow derived mesenchymal stromal cells from five adults (including middle‐aged to aged, GSM878095 ~ GSM878113). And the RNA‐seq data of adolescent hBMSCs (GSE160273) and hDPSCs (GSE148287) pulp derived mesenchymal stromal cells were analyzed. All data were normalized in the R computing environment using the DESeq2 package or limma package. All analyses were conducted in accordance with relevant regulations and guidelines. Differential expression analysis (DEA) was conducted and the common differentially genes were identified by Venn method.

In order to uncover the biological function alterations with cell senescence, GSEA was applied with genes pre‐ranked by their expression values< Gene set enrichment analysis: a knowledge‐based approach for interpreting genome‐wide expression profiles. >. GSVA package in R software was utilized to perform GSEA, using the predefined KEGG gene sets in Molecular Signature Database v7.2 < GSVA: gene set variation analysis for microarray and RNA‐seq data. + Molecular signatures database (MSigDB) 3.0.>. The enrichment score (ES value) was used for consensus clustering in R < Consensus clustering and functional interpretation of gene‐expression data. > .

GO enrichment analysis was performed using clusterProfiler. And the subcellular locations of the common DEGs were assessed based on Uniprot database. The roles of those common downregulated genes in DNA damage repair which were implied by GSVA, were further analyzed in Cytoscape<STRING v10: protein–protein interaction networks, integrated over the tree of life. > .

#### 5.2.11 Immunofluorescence staining

Cells were stained with anti‐YAP antibody overnight at 4°C. Samples were treated with fluorescent secondary antibodies following the manufacturer's protocol. DAPI was used to identify nuclear DNA. Images were captured on a Nikon Eclipse 300 fluorescence microscope (CompixInc).

#### 5.2.12 5‐Ethynyl‐2′‐deoxyuridine (EdU) incorporation labelling

EdU staining was conducted using an EdU imaging kit (RiboBio) according to the manufacturer's protocol. Cells were seeded on 96‐well plants overnight and then were incubated in medium containing 50 μM EdU for 3 h. After incubation, cells were subjected to EdU staining using Cell‐Light EdU Apollo567 In Vitro Kit (Ribobio) in accordance with its manuals. Images were captured with a Nikon Eclipse 300 fluorescence microscope (CompixInc).

#### 5.2.13 Immunoprecipitation

Cells of P3, P6, and P12 were harvested, lysed in lysis buffer (Beyotime) supplemented with a protease inhibitor cocktail (SAB), incubated on ice for 20 min, and cleared by centrifugation at 14,000 rpm at 4°C for 20 min. 500 μg of total protein lysate was incubated with agarose‐conjugated protein A/G beads (Beyotime) for 2 h at 4°C. Then the lysate was washed for five times with PBS at 4°C and centrifuged at 14,000 rpm at 4°C for 20 min. Finally, the lysate was subjected to immunoprecipitation with agarose (Beyotime)‐immobilized antibodies overnight at 4°C. The mix was washed for three times with lysis buffer at 4°C. The precipitated proteins were then detected by western blot.

#### 5.2.14 TEAD4 binding site analysis via ChIP‐seq

*TEAD4* binding site analysis of interest genes was performed in online ChIP‐seq database platform of UCSC Genome Brower (https://genome.ucsu.edu).

#### 5.2.15 ChIP‐qPCR

Cells at the confluence of 80% were prepared and subjected to ChIP respectively using Chromatin‐Prep‐Kit (MM_NF‐17‐10,461, Millipore) and HiSens‐Chromatin‐Immunoprecipitation‐Kit (MM_NF‐17‐375, Millipore, CA, USA) according to instruction manuals. After immunoprecipitation, chromatin was washed, eluted, reverse‐crosslinked and digested. Finally, purified DNA was analyzed by RT‐qPCR. Primers and antibodies used for ChIP‐qPCR were listed in Table S2.

### 5.3 Small molecules

TED347 was purchased from Selleck (USA, Texas, Cat# S8951). The storage concentration of TED347 was 10 mM, and the work concentration was 5 μM. 5‐FU was also purchased from Selleck (USA, Texas, Cat# S1209), and the storage concentration was 10 mM, work concentration was 5 μM. The schematic illustration of utilizing 5‐FU was showed in Figure 5.

#### 5.3.1 Quantification and statistical analysis

The statistical analysis of the RNA sequencing was calculated with ANOVA, whereas the statistical analysis of other experiments was carried out with Student's *t*‐test (two‐tailed) using Prism 5 (GraphPad Software). Error bars represent the standard deviation (SD). *p* values <0.05 were considered statistically significant. Significance labels in this paper were as follows: *p < 0.05; **p < 0.01; ***p < 0.001. All experiments were independently repeated by three times.

## AUTHOR CONTRIBUTIONS

F.Y. conceived the study; F.Y., L.Yao, and F.L. performed research; F.Y, L.Yao, and C.W. analyzed data; F.Y. wrote the manuscript; and L.Y. reviewed and edited the manuscript.

## CONFLICT OF INTEREST STATEMENT

The authors declare no conflict of interest.

## Supporting information


Appendix S1:

Data S1:

## Data Availability

The accession numbers for the RNA‐seq data reported in this paper are GSE160273 (hBMSCs) and GSE148287, GSE213339 (hDPSCs). All the original data and images are available from Fanyuan Yu upon request (fanyuan_yu@outlook.com).
