# Supplementary material for: Releasing YAP dysfunction‐caused replicative toxicity rejuvenates mesenchymal stem cells
Source: Aging Cell. 2023 Jun 20;22(9):e13913. doi: 10.1111/acel.13913 (PMC10497818; doi:10.1111/acel.13913)
Supplement: Supplementary file 1 — Appendix S1: [file ACEL-22-e13913-s001.pdf]

Figure S1

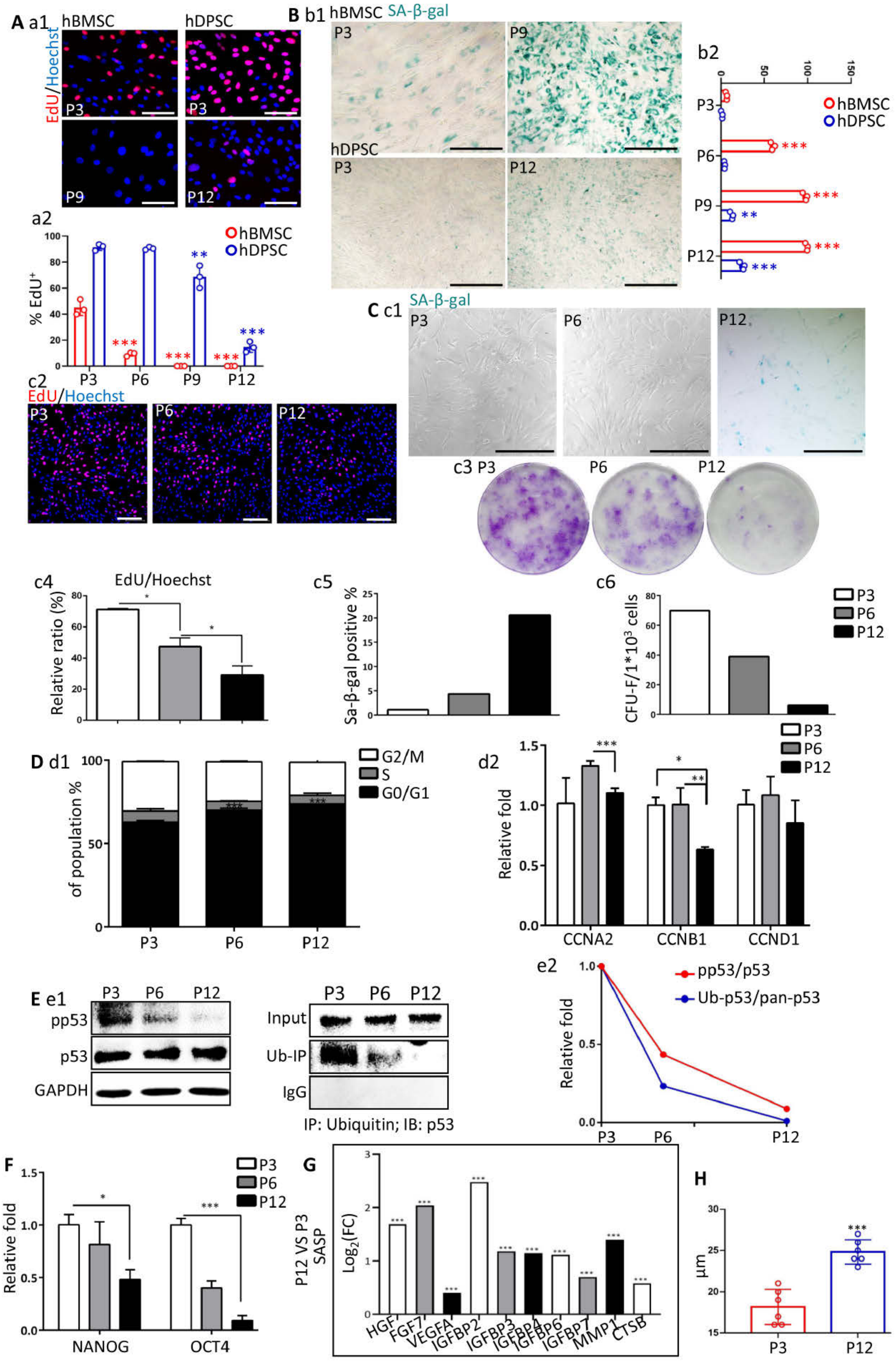

A a1 hBMSC hDPSC
EdU/Hoechst
P3 P3 P9 P12
a2
hBMSC
hDPSC
% EdU+
P3 P6 P9 P12
B b1 hBMSC SA-β-gal
P3 P9
hDPSC
P3 P12
b2
hBMSC
hDPSC
P3 P6 P9 P12
c2
EdU/Hoechst
P3 P6 P12
C c1 SA-β-gal
P3 P6 P12
c3 P3 P6 P12
c4 EdU/Hoechst
Relative ratio (%)
c5
Sa-β-gal positive %
c6
CFU-F/1*10³ cells
P3
P6
P12
D d1
of population %
G2/M
S
G0/G1
P3 P6 P12
d2
Relative fold
CCNA2 CCNB1 CCND1
E e1
P3 P6 P12
pp53
p53
GAPDH
Input
Ub-IP
IgG
IP: Ubiquitin; IB: p53
e2
pp53/p53
Ub-p53/pan-p53
Relative fold
P3 P6 P12
F
Relative fold
NANOG OCT4
G
P12 VS P3 SASP
Log2(FC)
HGF FGF7 VEGFA IGFBP2 IGFBP3 IGFBP4 IGFBP6 IGFBP7 MMP1 CTSB
H
µm
P3 P12

Figure S2

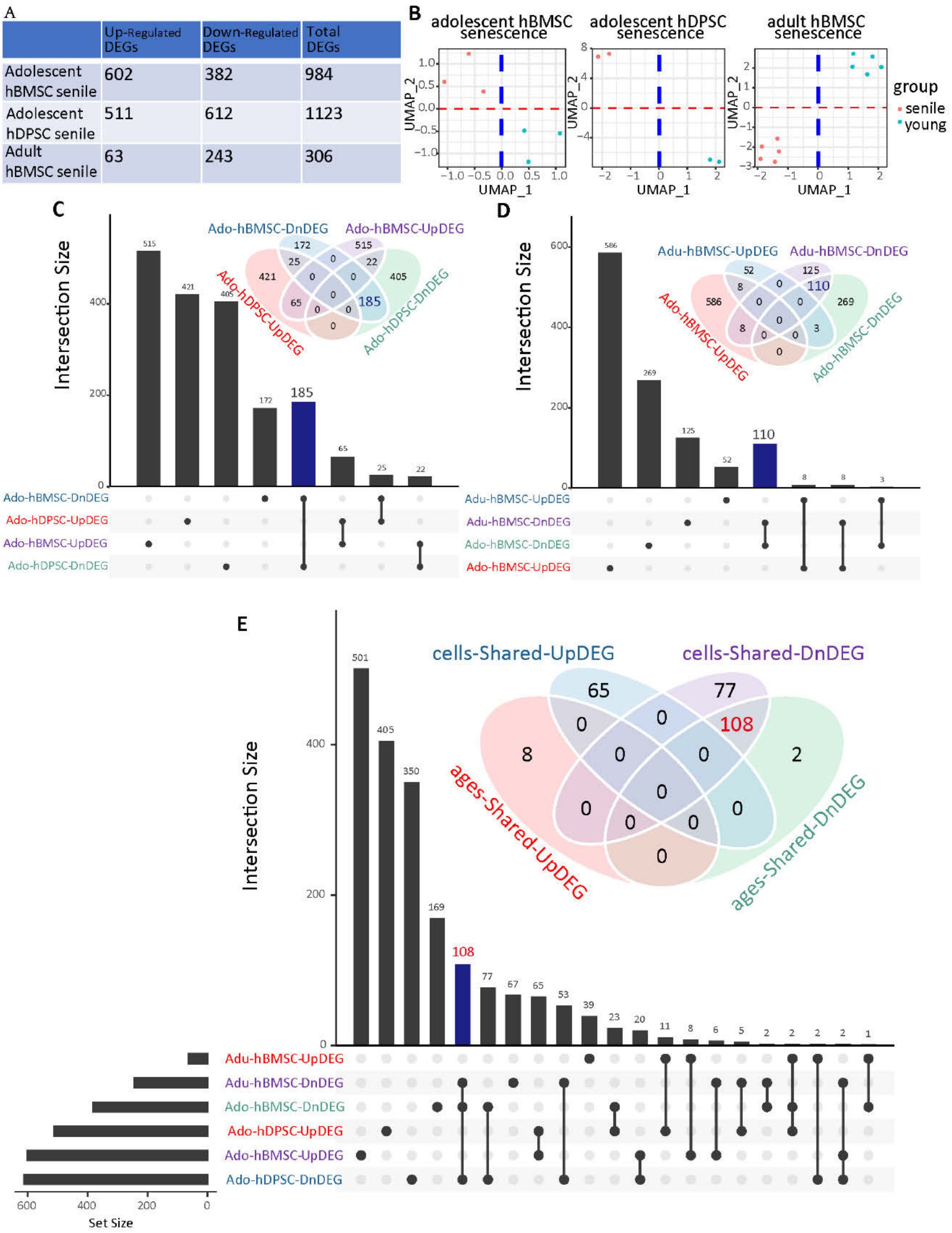
A
Up-Regulated DEGs
Down-Regulated DEGs
Total DEGs
Adolescent hBMSC senile 602 382 984
Adolescent hDPSC senile 511 612 1123
Adult hBMSC senile 63 243 306
B
adolescent hBMSC senescence
adolescent hDPSC senescence
adult hBMSC senescence
UMAP_1
UMAP_2
group
senile
young
C
Intersection Size
Ado-hBMSC-DnDEG
Ado-hBMSC-UpDEG
Ado-hDPSC-UpDEG
Ado-hDPSC-DnDEG
D
Adu-hBMSC-UpDEG
Adu-hBMSC-DnDEG
Ado-hBMSC-DnDEG
Ado-hBMSC-UpDEG
E
cells-Shared-UpDEG
cells-Shared-DnDEG
ages-Shared-UpDEG
ages-Shared-DnDEG
Adu-hBMSC-UpDEG
Adu-hBMSC-DnDEG
Ado-hBMSC-DnDEG
Ado-hDPSC-UpDEG
Ado-hBMSC-UpDEG
Ado-hDPSC-DnDEG
Set Size

Figure S3

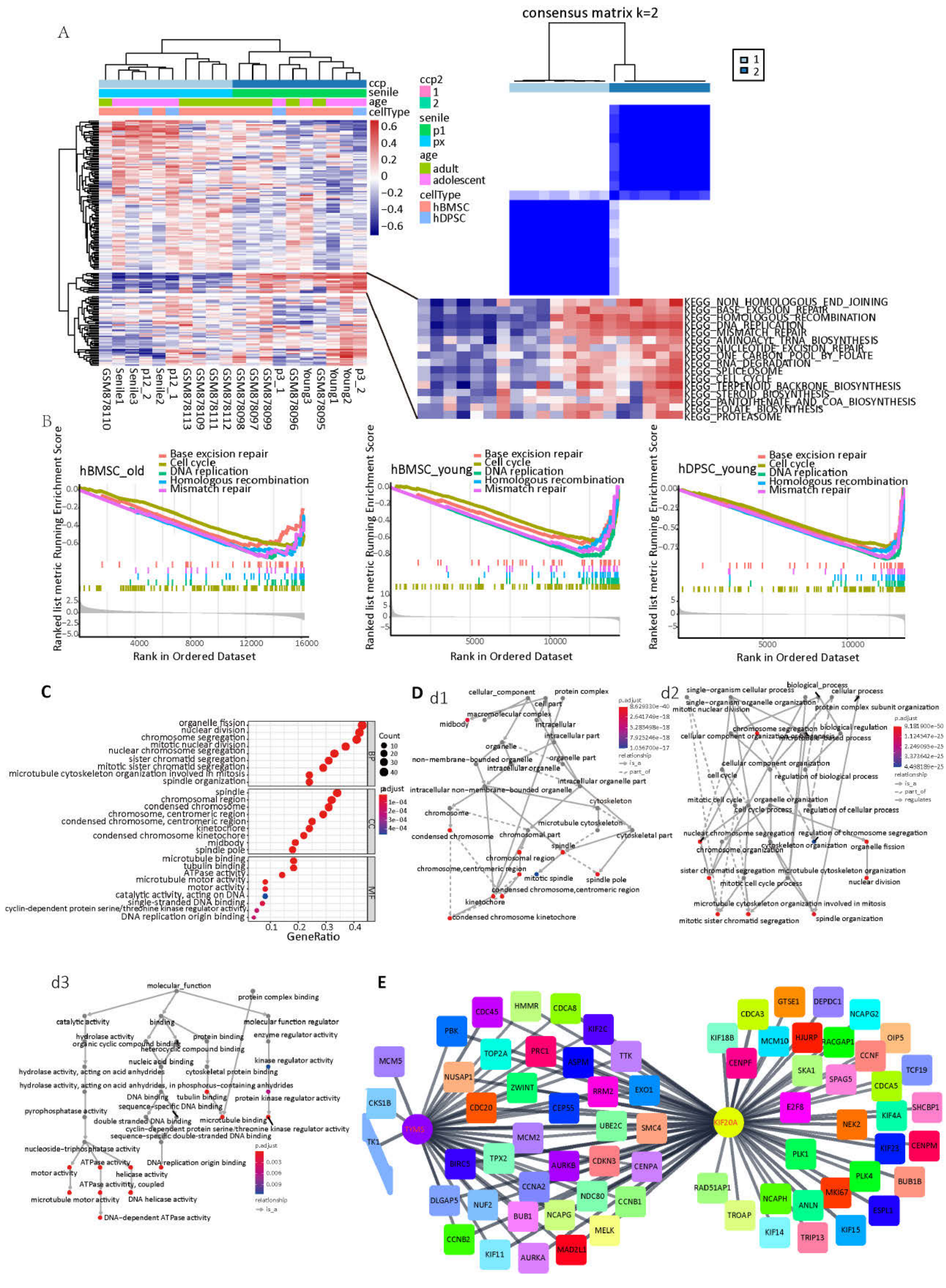
A
consensus matrix k=2
ccp
senile
age
cellType
ccp2
senile
p1
px
age
adult
adolescent
cellType
hBMSC
hDPSC
KEGG_NON_HOMOLOGOUS_END_JOINING
KEGG_BASE_EXCISION_REPAIR
KEGG_HOMOLOGOUS_RECOMBINATION
KEGG_DNA_REPLICATION
KEGG_MISMATCH_REPAIR
KEGG_AMINOACYL_TRNA_BIOSYNTHESIS
KEGG_NUCLEOTIDE_EXCISION_REPAIR
KEGG_ONE_CARBON_POOL_BY_FOLATE
KEGG_RNA_DEGRADATION
KEGG_SPLICEOSOME
KEGG_CELL_CYCLE
KEGG_TERPENOID_BACKBONE_BIOSYNTHESIS
KEGG_STEROID_BIOSYNTHESIS
KEGG_PANTOTHENATE_AND_COA_BIOSYNTHESIS
KEGG_FOLATE_BIOSYNTHESIS
KEGG_PROTEASOME
B
hBMSC_old
hBMSC_young
hDPSC_young
Base excision repair
Cell cycle
DNA replication
Homologous recombination
Mismatch repair
Running Enrichment Score
Ranked list metric
Rank in Ordered Dataset
C
organelle fission
nuclear division
chromosome segregation
mitotic nuclear division
nuclear chromosome segregation
sister chromatid segregation
mitotic sister chromatid segregation
microtubule cytoskeleton organization involved in mitosis
spindle organization
spindle
chromosomal region
condensed chromosome
chromosome, centromeric region
condensed chromosome, centromeric region
kinetochore
condensed chromosome kinetochore
midbody
spindle pole
microtubule binding
tubulin binding
ATPase activity
microtubule motor activity
motor activity
catalytic activity, acting on DNA
single-stranded DNA binding
cyclin-dependent protein serine/threonine kinase regulator activity
DNA replication origin binding
Count
p.adjust
GeneRatio
BP
CC
MF
D d1
d2
d3
E
TYMS
KIF20A

Figure S4

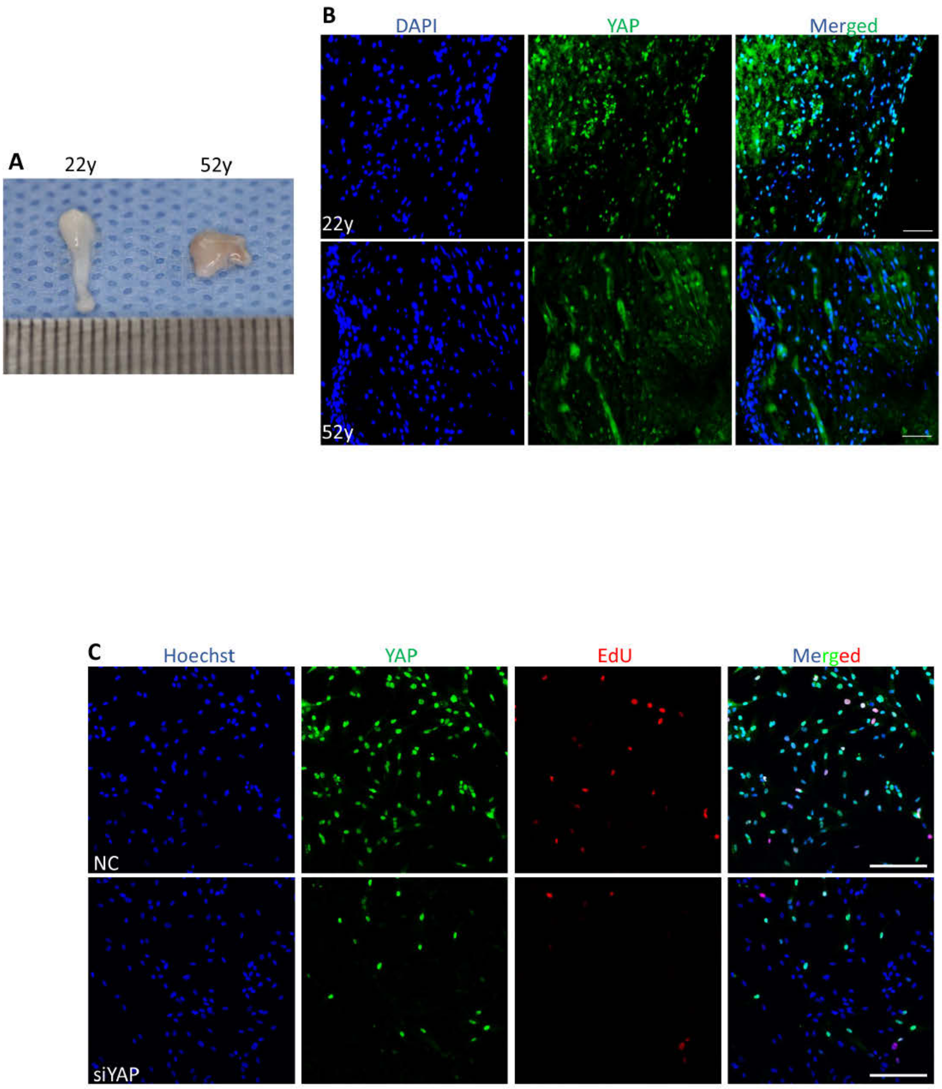
A
22y
52y
B
DAPI
YAP
Merged
22y
52y
C
Hoechst
YAP
EdU
Merged
NC
siYAP

Figure S5

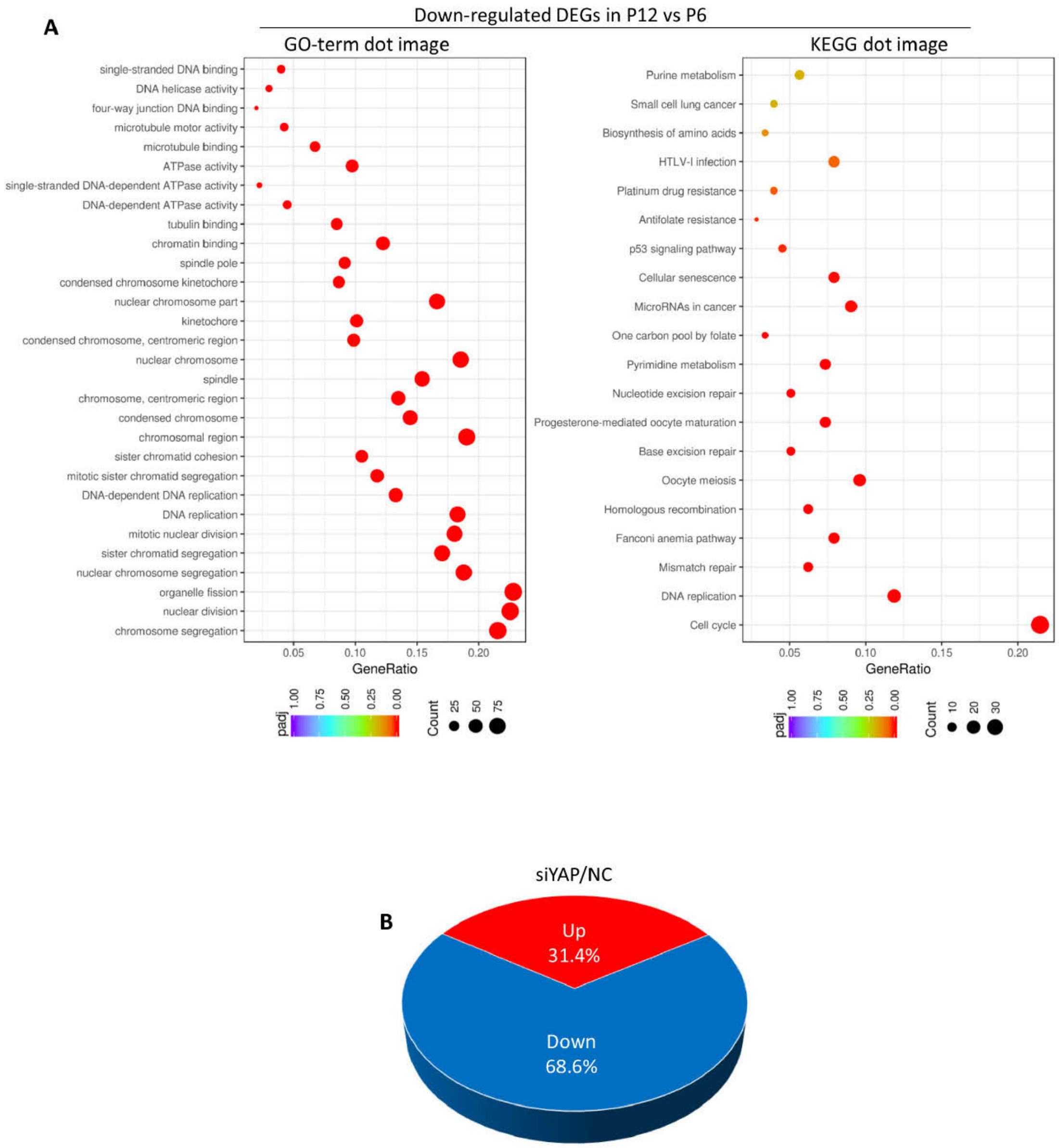
A
Down-regulated DEGs in P12 vs P6
GO-term dot image
single-stranded DNA binding
DNA helicase activity
four-way junction DNA binding
microtubule motor activity
microtubule binding
ATPase activity
single-stranded DNA-dependent ATPase activity
DNA-dependent ATPase activity
tubulin binding
chromatin binding
spindle pole
condensed chromosome kinetochore
nuclear chromosome part
kinetochore
condensed chromosome, centromeric region
nuclear chromosome
spindle
chromosome, centromeric region
condensed chromosome
chromosomal region
sister chromatid cohesion
mitotic sister chromatid segregation
DNA-dependent DNA replication
DNA replication
mitotic nuclear division
sister chromatid segregation
nuclear chromosome segregation
organelle fission
nuclear division
chromosome segregation
0.05 0.10 0.15 0.20
GeneRatio
padj 1.00 0.75 0.50 0.25 0.00
Count 25 50 75
KEGG dot image
Purine metabolism
Small cell lung cancer
Biosynthesis of amino acids
HTLV-I infection
Platinum drug resistance
Antifolate resistance
p53 signaling pathway
Cellular senescence
MicroRNAs in cancer
One carbon pool by folate
Pyrimidine metabolism
Nucleotide excision repair
Progesterone-mediated oocyte maturation
Base excision repair
Oocyte meiosis
Homologous recombination
Fanconi anemia pathway
Mismatch repair
DNA replication
Cell cycle
0.05 0.10 0.15 0.20
GeneRatio
padj 1.00 0.75 0.50 0.25 0.00
Count 10 20 30
siYAP/NC
B
Up
31.4%
Down
68.6%

923 **Figure S6**

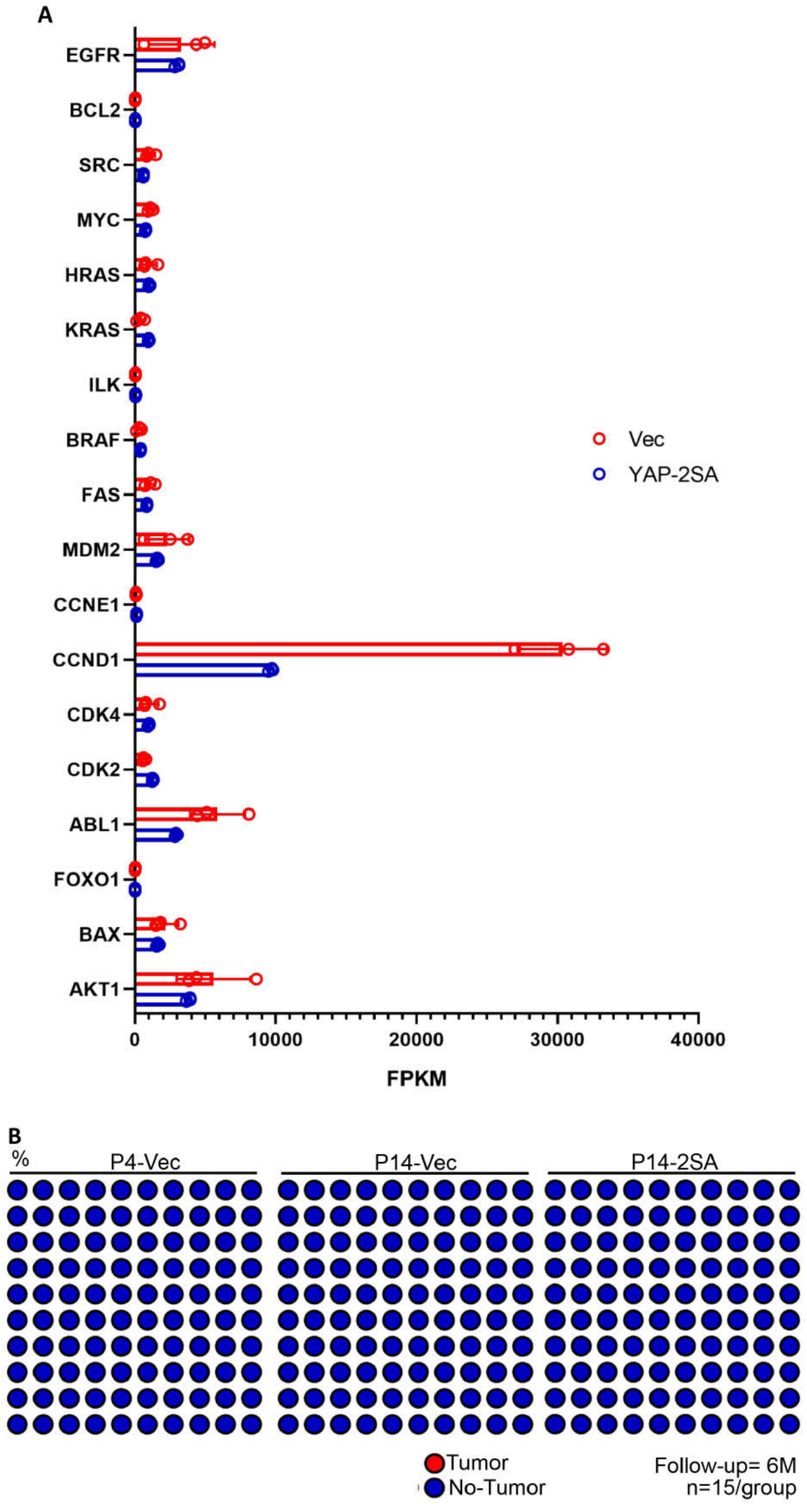

924

925

926

927

Figure S7

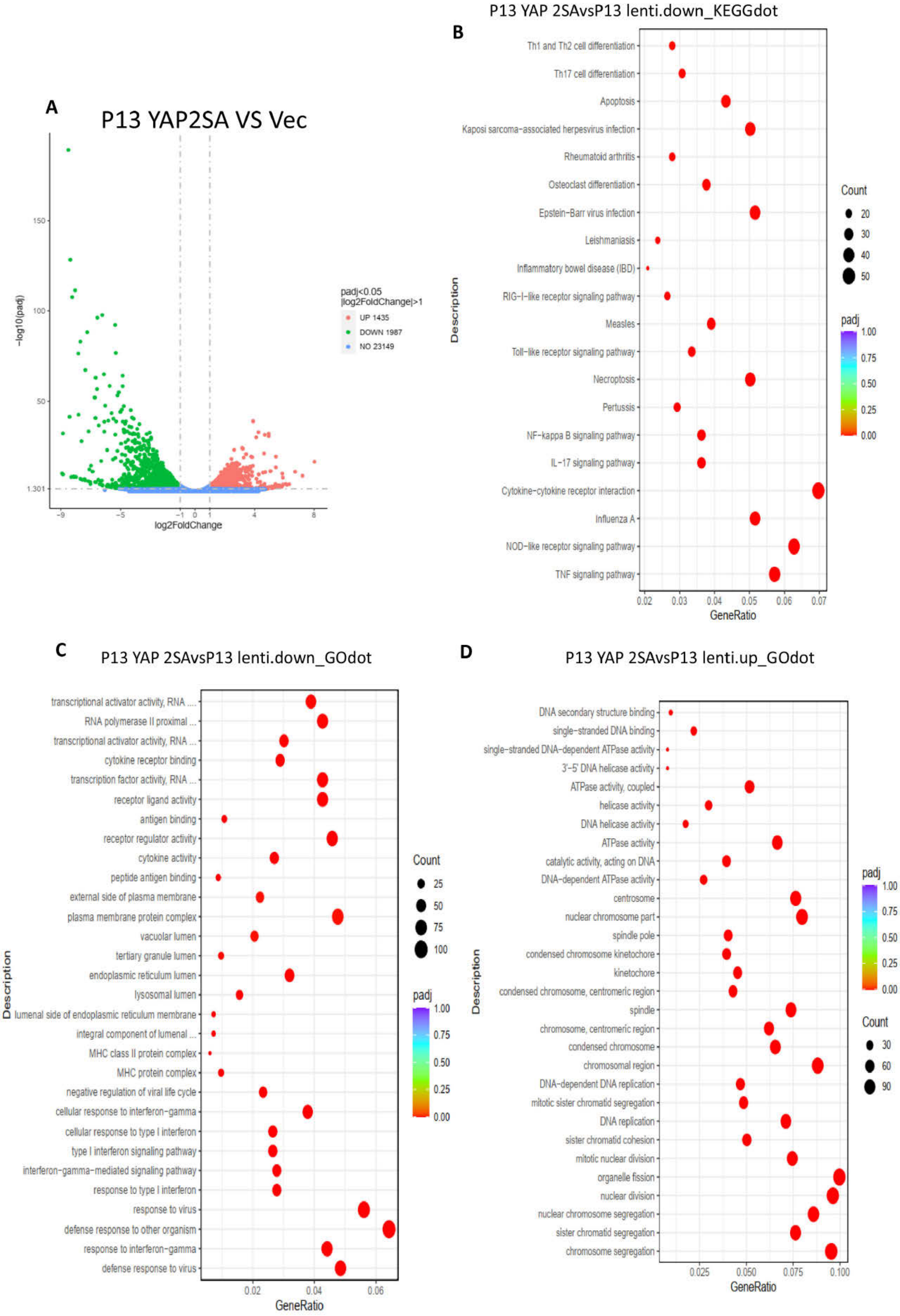
A
P13 YAP2SA VS Vec
-log10(padj)
log2FoldChange
padj<0.05 |log2FoldChange|>1
UP 1435
DOWN 1987
NO 23149
B
P13 YAP 2SAvsP13 lenti.down_KEGGdot
Th1 and Th2 cell differentiation
Th17 cell differentiation
Apoptosis
Kaposi sarcoma−associated herpesvirus infection
Rheumatoid arthritis
Osteoclast differentiation
Epstein−Barr virus infection
Leishmaniasis
Inflammatory bowel disease (IBD)
RIG−I−like receptor signaling pathway
Measles
Toll−like receptor signaling pathway
Necroptosis
Pertussis
NF−kappa B signaling pathway
IL−17 signaling pathway
Cytokine−cytokine receptor interaction
Influenza A
NOD−like receptor signaling pathway
TNF signaling pathway
Description
GeneRatio
Count
padj
C
P13 YAP 2SAvsP13 lenti.down_GOdot
transcriptional activator activity, RNA ...
RNA polymerase II proximal ...
transcriptional activator activity, RNA ...
cytokine receptor binding
transcription factor activity, RNA ...
receptor ligand activity
antigen binding
receptor regulator activity
cytokine activity
peptide antigen binding
external side of plasma membrane
plasma membrane protein complex
vacuolar lumen
tertiary granule lumen
endoplasmic reticulum lumen
lysosomal lumen
lumenal side of endoplasmic reticulum membrane
integral component of lumenal ...
MHC class II protein complex
MHC protein complex
negative regulation of viral life cycle
cellular response to interferon−gamma
cellular response to type I interferon
type I interferon signaling pathway
interferon−gamma−mediated signaling pathway
response to type I interferon
response to virus
defense response to other organism
response to interferon−gamma
defense response to virus
Description
GeneRatio
Count
padj
D
P13 YAP 2SAvsP13 lenti.up_GOdot
DNA secondary structure binding
single−stranded DNA binding
single−stranded DNA−dependent ATPase activity
3'−5' DNA helicase activity
ATPase activity, coupled
helicase activity
DNA helicase activity
ATPase activity
catalytic activity, acting on DNA
DNA−dependent ATPase activity
centrosome
nuclear chromosome part
spindle pole
condensed chromosome kinetochore
kinetochore
condensed chromosome, centromeric region
spindle
chromosome, centromeric region
condensed chromosome
chromosomal region
DNA−dependent DNA replication
mitotic sister chromatid segregation
DNA replication
sister chromatid cohesion
mitotic nuclear division
organelle fission
nuclear division
nuclear chromosome segregation
sister chromatid segregation
chromosome segregation
Description
GeneRatio
padj
Count

Figure S8

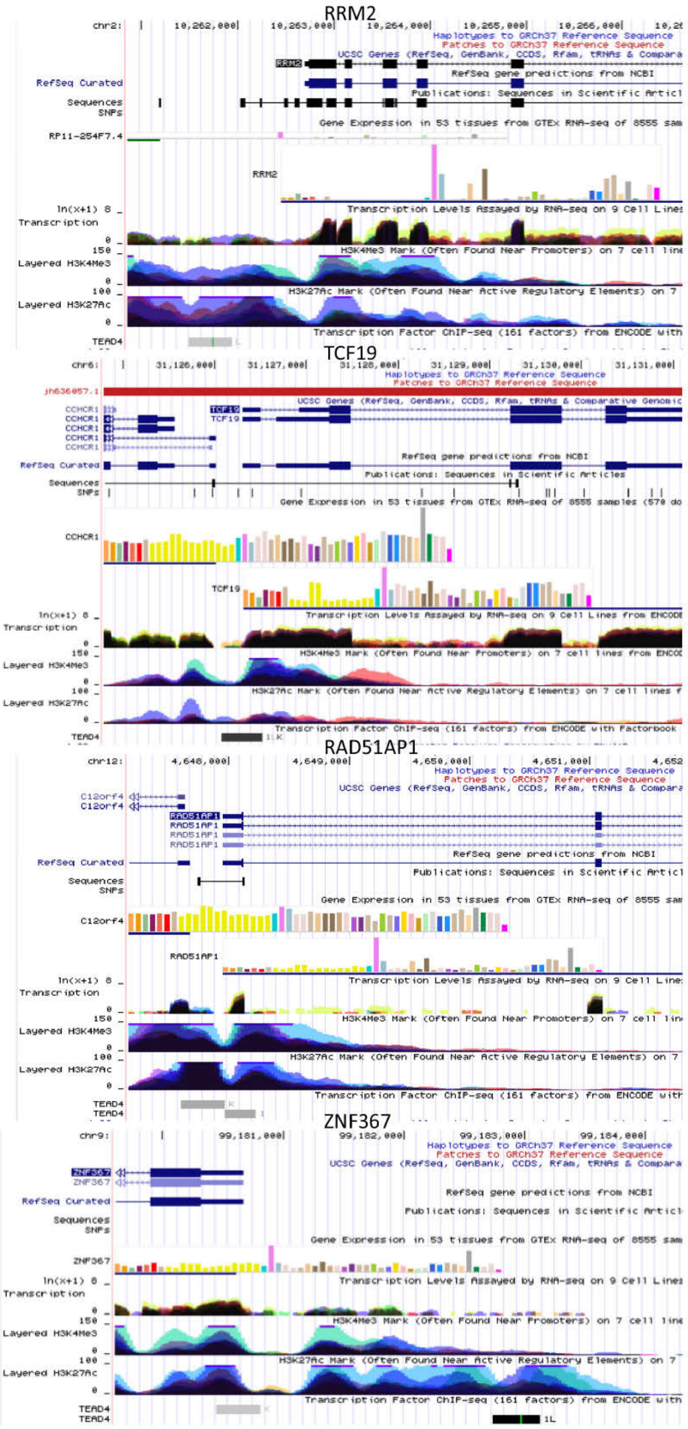

Figure S9

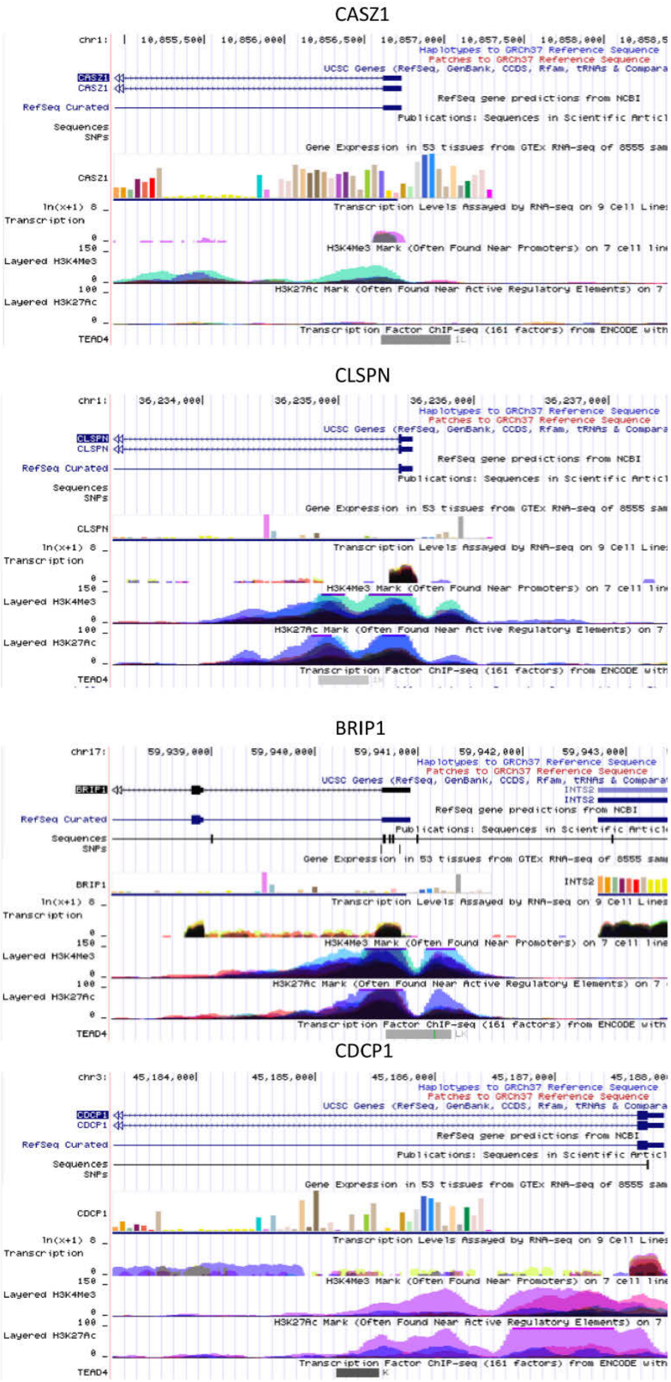

Figure S10

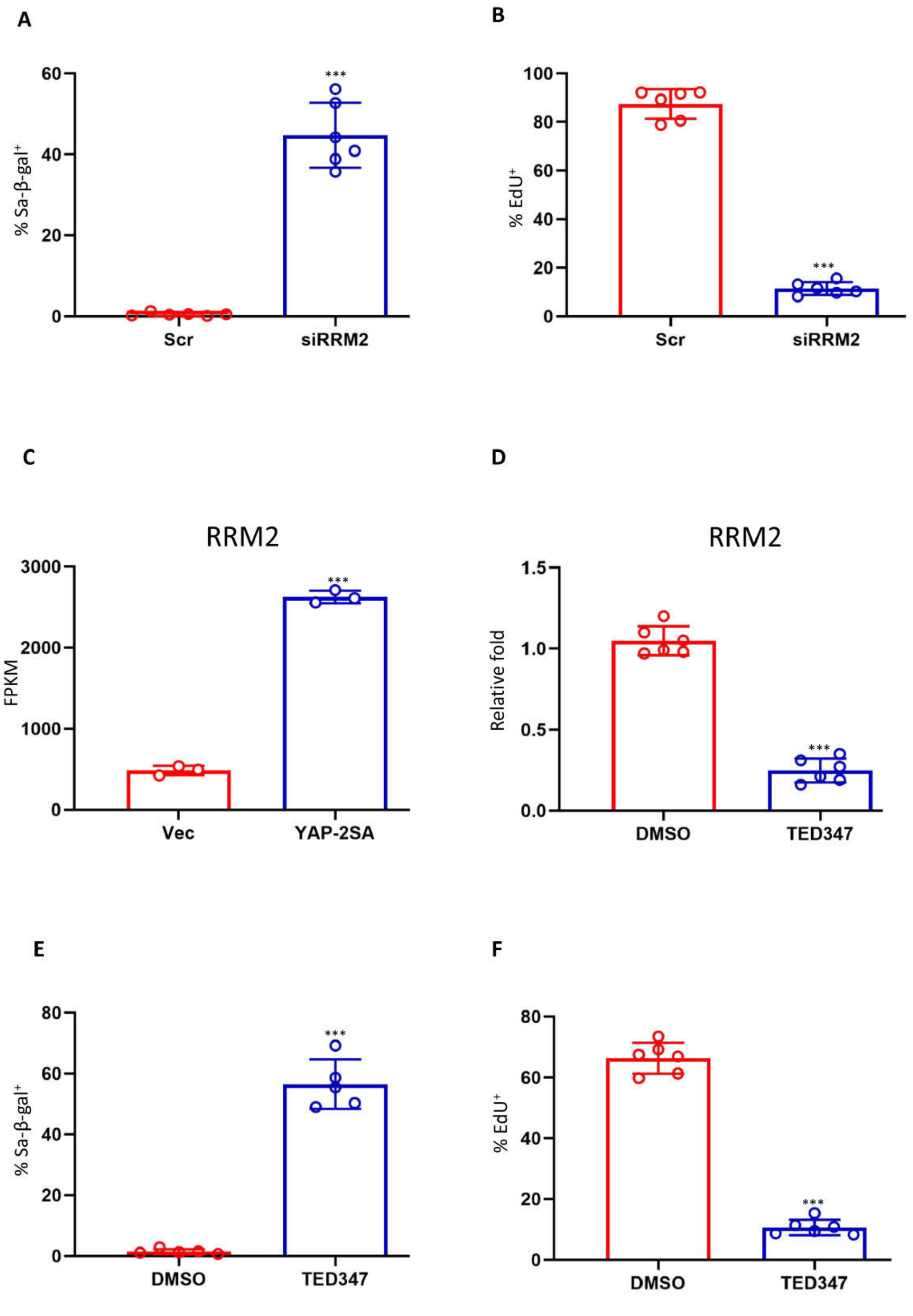
A
B
C
D
E
F
% Sa-β-gal⁺
% EdU⁺
Scr
siRRM2
RRM2
FPKM
Vec
YAP-2SA
Relative fold
DMSO
TED347
***

Table S1

Item	Provider	Cat no.	Application	Dilution
YAP	CST	14074	WB	1:2,000
			ChIP	1:50
YAP	Santa Cruz	sc-101199	WB	1:1000
			IF	1:1,00
Phospho-YAP (Ser127)	CST	4911	WB	1:1,000
GAPDH	CST	5174S	WB	1:10,000
ATR	CST	13934S	WB	1:1,000
RAD51	CST	8875S	WB	1:1,000
NBS	CST	14956T	WB	1:500
p-NBS	CST	3001T	WB	1:500
MRE11	CST	4847T	WB	1:1,000
Phospho-Histone H2A.X (Ser139)	CST	9718S	WB	1:1,000
			IF	1:200
OPG	Invitrogen	PA5-86053	IF	1:150
p-MRE11	CST	4859T	WB	1:1,000
p-ATM	CST	5883T	WB	1:1,000
p-ATR	CST	2853T	WB	1:1,000
RAD50	HuaBio	EMI707-66	WB	1:1,000
ATM	HuaBio	ET1606-20	WB	1:1,000
DNA-PK	HuaBio	M1204-6	WB	1:1,000
RRM2	HuaBio	ET1705-62	WB	1:1,000
p53	SantaCruz	sc-47698	WB	1:1,000
p-p53	SantaCruz	sc-377567	WB	1:1,000
β-Actin	SAB	21338	WB	1:5000
Ubiquitin	Abcam	ab19247	IP	1:50
alpha Tubulin	Abcam	ab52866	WB	1:10,000
Goat anti-rabbit IgG(HRP)	Abcam	ab6721	WB	1:10,000
Goat anti-mouse IgG (HRP)	Abcam	ab6789	WB	1:10,000
Goat anti-Mouse IgG-H&L (Alexa Fluor® 488)	Abcam	ab150113	IF	1:500
CY3 Conjugated	BOSTER	BA1031	IF	1:200

AffiniPure Goat Anti-mouse IgG (H+L)				
normal rabbit IgG	CST	2729	ChIP	1:50

Table S2

RT-qPCR primers

Gene	Sequence (5’~3’)
GAPDH(F)	CTCCTGCACCACCAACTGCT
GAPDH (R)	GGGCCATCCACAGTCTTCTG
CYCLIN A2-(F)	TGGTGGTCTGTGTTCTGTGA
CYCLIN A2-(R)	TGCCAGTCTTACTCATAGCTGA
YAP(F)	CCTCGTTTTGCCATGAACCAG
YAP(R)	GTTCTTGCTGTTTCAGCCGCAG
CYCLIN D1-(F)	GTTGCAAAGTCCTGGAGCCT
CYCLIN D1-(R)	CACAGGAGCTGGTGTTCCAT
OCT4(F)	GACAACAATGAGAACCTTCAGGAGA
OCT4(R)	TTCTGGCGCCGGTTACAGAACCA
NANOG(F)	ATAGCAATGGTGTGACGCAG
NANOG(R)	GATTGTTCCAGGATTGGGTG
CYCLIN B1(F)	AAGAGCTTTAAACTTTGGTCTGGG
CYCLIN B1(R)	CTTTGTAAGTCCTTGATTTACCATG
CTGF(F)	CCAATGACAACGCCTCCTG
CTGF(R)	TGGTGCAGCCAGAAAGCTC
CYR61(F)	AGCCTCGCATCCTATACAACC
CYR61(R)	TTCTTTCACAAGGCGGCACTC
ANKRD1(F)	AGTAGAGGAACTGGTCACTGG
ANKRD1(R)	TGGGCTAGAAGTGTCTTCAGAT
BRCA2(F)	TGATCCAAAGGGTCCCAAAGTTTC
BRCA2(R)	TTCACAGCTTTTTGCAGAGCCTCACA
CASZ1(F)	CAAAACAGACTCCATCACCACG
CASZ1(R)	GTGCTGGCTGCCCGAGAAC
MCUB(F)	CCTCCGCCCCAGGTTTT

MCUB(R)	GTGTTACCAAGGGAAGGCCA
RHBDF2(F)	GGCAAACTCAGACTCGAAG
RHBDF2(R)	CGCTGACTCCAAACCACTG
CLSPN (F)	ATG ATT CCC AGA TGG ACT TG
CLSPN (R)	AGC CAC TGC TCT CGT TCA AT
DTL(F)	AAGTGCTTCAAAGAATGGATGGCTC
DTL(R)	GAATATCCAAGTTTTCGAGTGCTGCT
RAD51AP1(F)	CGCCTTGGCTTGTCCAGAT
RAD51AP1(R)	GGTGCTAGTGGCATTTGGATG
FANCI(F)	AGCATCTCACTGCTGTGCTT
FANCI(R)	GGACCTCTGAAATTGCCGGA
RRM2(F)	ACGGAGCCGAAAACTAAAGCAGCT
RRM2(R)	AGAGTCCACCTCCTCGGCG
GGT5(F)	ACCTGCAGTGAGTGGAGACA
GGT5(R)	GACCCCTGGGCTTTGTTGAT
TCF19(F)	CGTGTAGACAAAGCCCCACT
TCF19(R)	CAGGCCACTGTCTCTTCCTG
CDC25B(F)	CACGCCCGTGCAGAATAAGC
CDC25B(R)	ATGACTCTCTTGTCCAGGCTACAGG
CDCA5(F)	GACGCCAGAGACTTGGAAATG
CDCA5(R)	GGACCTCGGTGAGTTTGGAG
OAS3(F)	GGCTGGAACTCCTGACTGTG
OAS3(R)	GATGATAGGCCTGGGCTTCTG
MASTL(F)	TATGCCACATCAGCAGACCC
MASTL(R)	CAAGCAAACTCCAAGTGCCC
CDCP1(F)	CGGGGCAGAAGCTTTTGAGA
CDCP1(R)	CAGGCGAGGGATGGAAAACT
RMI2(F)	GGTGATGGGAGTGGTTCAGG
RMI2(R)	TCTTCTACCTCCAGTTCCCACA
FAM111B(F)	TCTGGGTAGGCGGTATGCTA
FAM111B (R)	TTACCCACTGTGCCTCCTCT
FAM111A(F)	CTTCACAAAAAGGGGCGCAA
FAM111A(R)	ATCAACTGGCTGGGTGCTTT

ASF1B (F)	GTTTGTCTTTCAGGCCGACG
ASF1B (R)	ACGTAGTAGCCCACTCGGAT
BRIP1(F)	GCAGATGAGGGCGTAAGTGA
BRIP1(R)	AACTTTGCAGCCAGAGTGGT
CDC25A(F)	GAAGAATACATTCCCTACCTCAGA
CDC25A(R)	TGAGGAAAGCATCCGAGCTG
CDT1(F)	TTACCAGCTCACCATCGAGC
CDT1(R)	CCTTGTGGTGCTCCTTGACA
CEP72(F)	TTTGGATCTCTCGCGCAACT
CEP72(R)	ACACTTCTGCCAACGAGGAG
FAAP24(F)	ACGTGCCTTTGGGGCATATT
FAAP24(R)	AGTCTGGTGTCAAGCCATCC
L2HGDH(F)	AAGACCGCTGTGTGGAGGTA
L2HGDH(R)	CCTGGGCAAATGACAAAGCC
NEIL3(F)	GCAGTAAACACAACCGCCTC
NEIL3(R)	TTTCATGGTGGAACGCTTGC
ORC6(F)	GTCCAGTCTTCCCCAGACAC
ORC6(R)	CATTTCCTTTGCTGGGGCTTC
POLA2(F)	CCTCATCATCCCGTCAGAGC
POLA2(R)	CCGGCCTCCTAAGGTAGAGT
CDC6(F)	CTGGATGTTTGCAGGAGAGC
CDC6(R)	TTGTGCTCCTTCTTGGCTCA
ZNF367(F)	GTGAGAGGCCCTATCTGTGTG
ZNF367(R)	GCGGTTTGCATGGGTGAATC
CYCLIN E1(F)	CCCCATCATGCCGAGGGA
CYCLIN E1(R)	CTCGCCGTCCTGTCGATTTT

ChIP-qPCR primers

Gene	Sequence (5'~3')
RRM2 (F)	TTATGACCACCTTCGGCACG
RRM2 (R)	AAGGTTAGCGCGACTTCCAA

siRNAs for RRM2 (5'~3')

RRM2-HOMO-667	GGAGCGAUUUAGCCAAGAATT<br>UUCUUGGCUAAAUCGCUCCTT

RRM2-HOMO-480	CCAUCGAGUACCAUGAUAUTT<br>AUAUCAUGGUACUCGAUGGTT
RRM2-HOMO-1179	GCACUCUAAUGAAGCAAUATT<br>UAUUGCUUCAUUAGAGUGCTT
