# Supplementary material for: Releasing YAP dysfunction‐caused replicative toxicity rejuvenates mesenchymal stem cells
Source: Aging Cell. 2023 Jun 20;22(9):e13913. doi: 10.1111/acel.13913 (PMC10497818; doi:10.1111/acel.13913)
Supplement: Supplementary file 2 — Data S1: [file ACEL-22-e13913-s002.docx]

Figure S1. RT emerges upon in vitro expansion of dental MSCs. (A) Representative EdU incorporation images (a1) and its statistic data (a2). n = 3 per group. Cells used in this study were hDPSCs, otherwise specifically indicated. (B) Representative senescence-associated β-gal (SA-β-gal) images (b1) and its statistic data (b2). n = 3 per group. (C) Representative SA-β-gal (c1), EdU incorporation (c2), and colony-forming units (CFU) (c3) images and their statistic data respectively (c4-6). (D) (d1) The statistical data of flowcytometry (FCT) of cell cycle, and (d2) RT-qPCR data. n = 5 per group. (E) Representative western blotting data of immunoprecipitation (e1) and its statistics (e2). IP, immunoprecipitation; IB, immunoblotting. (F) RT-qPCR data. n = 3 per group. (G) Statistical data of RNA-seq between P12 versus P3 of hDPSCs. FC, fold change. (H) Quantification of cell size. *, p < 0.05; **, p < 0.01; ***, p < 0.001. Scale bars, 20 μm. All experiments were technically replicated for triple times.
Figure S2. RT owns core transcriptomic signature of 108 down-regulated genes for cell cycle and DNA damage regulation. (A) The numbers of up-regulated genes (UpDEGs) and down-regulated genes (DnDEGs) in the three datasets. DEGs, deferentially expressed genes. (B) the UMAP cluster plot of samples in the three datasets, showing the clustering and separation of samples. Dots in red represent senile cell samples while dots in blue represent young cell samples. (C-D) The upset plot of intersections of DEGs between different cell types (C) and between different host age groups (D), showing the down-regulated DEGs was the major category shared in groups (blue bar). (E) The upset plot of shared intersected DEGs between cell types and age groups, showing the 108 DEGs shared were all the down-regulated DEGs. n = 3 per group, and all experiments were technically replicated for triple times.
Figure S3. The core transcriptomic alteration of RT consists of cell cycle regulation and genome instability. (A) Heatmap of KEGG pathway scores based on GSVA algorithms. Pathways were clustered into three types, one of which was characterized by significant down regulation in senile groups, including NON HOMOLOGOUS END JOINING, BASE EXCISION REPAIR, HOMOLOGOUS RECOMBINATION, CELL CYCLE, MISMATCH REPAIR, NUCLEOTIDE EXCISION REPAIR. (left). Two category classification cells samples depending on the pathway scores by R package ConsensusClusterPlus, were identical to sample types grouped by senile or young, indicating the similarity of biological process underlying senescence in different stromal stem cells and various host age groups (right). (B) GSEA plot of DNA damaging repair pathways during cell cycle and DNA replication, showing its enrichment in young stem cells. (C) GO-term bubble plot of the 108 core down-regulated DEGs for RT. (D) GO-term hierarchy diagram of the 108 core down-regulated DEGs for RT. (E) Protein–protein interaction network of the 108 common down-regulated DEGs.
Figure S4. YAP dysfunction also occurs in chronological aging. (A) The photos of isolated human dental pulp tissues. (B) Representative IF images of YAP. (C) Representative IF images of YAP and EdU incorporation after silencing YAP in hDPSCs at P4. Scale bars, 20 μm.
Figure S5. RNA-seq data of P12 versus P6 and siYAP versus control. (A) GO-term and KEGG analysis of RNA-seq data for P12 versus P6. (B) Statistical summary of DEGs after knocking down YAP.
Figure S6. YAP 2SA in hDPSCs is free of risks of tumorigenesis. (A) RNA-seq data showing the crucial oncogenes. (B) Statistical data of the prevalence of tumors after hDPSCs transplantation.
Figure S7. RNA-seq analysis for YAP 2SA. Volcano plot showing the DEGs between YAP 2SA and control group (Vec) at P13. (B-D) KEGG (B) and GO-term analysis of DEGs between YAP 2SA and control group (Vec) at P13.
Figure S8-S9. Screening candidate downstream of YAP/TEAD4 complex. UCSC genome browser images presenting the top-reduced genes which owned high enrichment of TEAD4 within their H3K27Ac-enriched transcription start sequences (TSS), namely loose chromatin zones, according to ChIP-seq data.
Figure S10. Statistical data for Figure 6. (A) Statistical data of SA-β-gal of Figure 4h. (B) Statistical data of EdU of Figure 4I. (C) RNA-seq data of RRM2 in P13 hDPSCs between YAP 2SA and control group. (D) RT-qPCR data of RRM2 after using TED347 at P4. (E) Statistical data of SA-β-gal of Figure 4k. (F) Statistical data of EdU of Figure 4M. ***, p < 0.001. All experiments were technically replicated for triple times.

Table S1. The list of key material resources.

Table S2. The list of RT-qPCR and ChIP-qPCR primers.
